# ZSWIM4 inhibition improves chemosensitivity in epithelial ovarian cancer cells by suppressing intracellular glycine biosynthesis

**DOI:** 10.1186/s12967-024-04980-8

**Published:** 2024-02-21

**Authors:** Kunxiang Gong, Yinger Huang, Yanqin Zheng, Wenbo Hao, Kun Shi

**Affiliations:** 1 Institute of Reproductive Health and Perinatology, Guangzhou Women and Children’s Medical Center, Guangzhou Medical University, Guangzhou, 510623 China; 2 Department of Gynecology and Obstetrics, Guangzhou Women and Children’s Medical Center, Guangzhou Medical University, Guangzhou, 510623 Guangdong China; 3 School of Traditional Chinese Medicine, Southern Medical University, Guangzhou, 510515 Guangdong China; 4 Institute of Antibody Engineering, School of Laboratory Medicine and Biotechnology, Southern Medical University, Guangzhou, 510515 Guangdong China

**Keywords:** Epithelial ovarian cancer, Carboplatin, ZSWIM4, Chemosensitivity, FOXK1, Glycine biosynthesis

## Abstract

**Background:**

Zinc finger SWIM-type containing 4 (ZSWIM4) induces drug resistance in breast cancer cells. However, its role in epithelial ovarian cancer (EOC) remains unknown. In this study, we aimed to investigate the clinical significance of ZSWIM4 expression in EOC and develop new clinical therapeutic strategies for EOC.

**Methods:**

ZSWIM4 expression in control and EOC tumor tissues was examined using immunohistochemistry. Lentiviral transduction, Cell Counting Kit-8 assay, tumorsphere formation assay, flow cytometry, western blotting, and animal xenograft model were used to assess the role of ZSWIM4 in chemotherapy. Cleavage Under Targets and Tagmentation (CUT&Tag) assays, chromatin immunoprecipitation assays, and luciferase reporter assays were used to confirm FOXK1-mediated upregulation of ZSWIM4 expression. The mechanism by which ZSWIM4 inhibition improves chemosensitivity was evaluated using RNA-sequencing. A ZSWIM4-targeting inhibitor was explored by virtual screening and surface plasmon resonance analysis. Patient-derived organoid (PDO) models were constructed from EOC tumor tissues with ZSWIM4 expression.

**Results:**

ZSWIM4 was overexpressed in EOC tumor tissues and impaired patient prognoses. Its expression correlated positively with EOC recurrence. ZSWIM4 expression was upregulated following carboplatin treatment, which, in turn, contributed to chemoresistance. Silencing ZSWIM4 expression sensitized EOC cells to carboplatin treatment in vitro and in vivo. FOXK1 could bind to the GTAAACA sequence of the *ZSWIM4* promoter region to upregulate *ZSWIM4* transcriptional activity and FOXK1 expression increased following carboplatin treatment, leading to an increase in ZSWIM4 expression. Mechanistically, ZSWIM4 knockdown downregulated the expression of several rate-limiting enzymes involved in glycine synthesis, causing a decrease in intracellular glycine levels, thus enhancing intracellular reactive oxygen species production induced by carboplatin treatment. Compound IPN60090 directly bound to ZSWIM4 protein and exerted a significant chemosensitizing effect in both EOC cells and PDO models.

**Conclusions:**

ZSWIM4 inhibition enhanced EOC cell chemosensitivity by ameliorating intracellular glycine metabolism reprogramming, thus providing a new potential therapeutic strategy for EOC.

**Supplementary Information:**

The online version contains supplementary material available at 10.1186/s12967-024-04980-8.

## Introduction

Among all gynecological malignancies, ovarian cancer (OC) has the highest mortality rate [1]. Among all OC types, epithelial ovarian cancer (EOC) is the most common, accounting for over 90% of all cases [2]. Over the past three decades, platinum-based therapy has represented the most administered treatment for patients with EOC [3]. However, owing to disease heterogeneity, many patients experience recurrence, portending an extremely low survival rate [4]. The mechanisms of EOC chemoresistance are diverse, including increased intracellular drug pumping [5], deactivation of tumor suppressor gene (*retinoblastoma* and *phosphatase and tensin homolog*) transcription [6], and reverse mutations in breast cancer (*BRCA*)*1* and *BRCA2* [7]. However, the benefits of current treatment strategies for patients with EOC remain limited, with a poor overall prognosis. Therefore, an urgent need exists for the identification of molecular markers affecting recurrence in patients with EOC and the development of novel therapeutic targets to improve their clinical prognosis.

Zinc finger switching (SWI)2/Sucrose NonFermenting (SNF)2 and MuDR (SWIM) type-containing (ZSWIM) family members are named due to the uniqueness of their structural domains [8]. Reports suggest that ZSWIM proteins have strong regulatory functions in cells, whether they bind to deoxyribonucleic acid (DNA) to regulate gene transcription [9] or play a role in ubiquitination to affect target protein expression [10, 11]. Increased ZSWIM4 expression is correlated with the high clinical stage of colon adenocarcinoma [12], and activated transforming growth factor beta (TGF-β) signaling results in the upregulation of ZSWIM4 in breast cancer cells [13]. Our previous study showed that ZSWIM4 could mediate resistance to apoptosis induced by a non-receptor-type tyrosine-protein Janus kinase 2 (JAK2) inhibitor by upregulating vitamin D receptor levels in breast cancer cells. Furthermore, ZSWIM4 knockdown could counteract resistance to JAK2 inhibitors [14]. These findings indicate that high ZSWIM4 expression in tumor cells could contribute to drug resistance. However, its function in ovarian malignancy has rarely been studied. Hence, clarifying the specific role of ZSWIM4 in EOC is imperative for the clinical treatment of this disease.

The process by which cells promote growth and proliferation regulating their metabolism is known as metabolic reprogramming [15]. Tumor cells adaptively change their metabolic processes to meet their required proliferation capacity; therefore, metabolic reprogramming is considered a new tumor marker [16]. Recently, increasing evidence has shown that repression of aberrant metabolism within tumor cells enhances EOC cell chemosensitivity. For example, inhibiting fatty acid oxidation enhances the therapeutic efficacy of platinum drugs in EOC [17]. Moreover, active amino acid metabolism in EOC cells is crucial for maintaining stem cell-like characteristics [18] and drug resistance [19]. Meanwhile, one-carbon unit pathway metabolism reportedly plays an important role in cancer treatment [20]. Targeting the key enzymes involved in serine and glycine biosynthesis elicits a synergistic effect with Olaparib in EOC cells [21]. However, the abnormal metabolism of reduced glutathione (GSH) in EOC cells is a vital driver of platinum resistance [22], and GSH inhibition enhances EOC cell sensitivity to platinum drugs [23]. Although several studies have shown that metabolic reprogramming contributes to the therapeutic resistance of EOC, the specific drivers and mechanisms underlying its regulation remain unknown. Uncovering the molecular mechanisms by which tumor cells regulate metabolic reprogramming to exhibit chemoresistance could provide a more definitive theoretical basis for overcoming EOC chemoresistance.

In this study, we report that ZSWIM4 is highly expressed in EOC tumor tissues and is responsible for chemoresistance in EOC cells. Moreover, ZSWIM4 inhibition improves EOC cell chemosensitivity by regulating intracellular glycine metabolism. Collectively, our study provides a novel target for overcoming chemotherapy resistance in EOC cells.

## Materials and methods

### Ethics statement

The acquisition and use of human specimens were in concordance with the International Ethical Guidelines for Research Involving Human Subjects, as stated in the Helsinki Declaration, and were approved by the Medical Ethics Committee of Guangzhou Women and Children’s Medical Center (2022416B01). All the tissue samples were collected after obtaining signed informed consent from the participants. The use of ovarian cancer tissue microarray (TMA) was approved by the Ethics Committee of Shanghai Outdo Biotech Company (SHYJS-CP-1804010). All experiments involving animals were conducted according to the ethical policies and procedures approved by the Southern Medical University Animal Care and Use Committee (L-2020163).

### Cell culture and sample collection

Three human EOC cell lines (OVCAR8, SKOV3 and ES-2) were obtained from Procell Life Science & Technology (Wuhan, China). Normal human ovarian surface epithelial cells (IOSE-80) were obtained from iCell Bioscience Inc. (Shanghai, China). The cells were tested and confirmed to be free of *Mycoplasma*. The cell passage number was no more than 20. OVCAR8 cells were cultured in Roswell Park Memorial Institute 1640 medium (Gibco, Grand Island, NY, USA) supplemented with 10% fetal bovine serum (FBS) (Gibco). SKOV3 cells were cultured in McCoy’s 5A (Gibco) plus 10% FBS. Carboplatin (CBP)-resistant SKOV3 cells (SKOV3/CBP) were generated from parental SKOV3 cells by treating them with gradually ascended concentrations of CBP. All cells were cultured at 37 °C with 5% CO_2_.

We collected 15 paraffin-embedded human EOC tissue sections and 15 paraffin-embedded corresponding normal sections, including 10 paired samples and two fresh human EOC tumor tissue samples, for this study. All samples were obtained from September 2021 to June 2023 from patients from Guangzhou Women and Children's Medical Center, Guangzhou, China diagnosed with EOC who had not received chemotherapy before surgery. The ovarian cancer tissue microarray (TMA) (Cat. HOvaC151Su01) was provided by Outdo Biotech (Shanghai, China). Excluding the exfoliated samples and necrotic tissues, we included 117 cases of primary lesions in our analysis, including 114 cases of EOC and three cases of tumors from germ cells. Our analysis was based on the ZSWIM4 immunohistochemical score in the 114 cases.

### Reagents

Antibodies against cysteine-aspartic protease (caspase)-3 (Cat. #14,220), cleaved caspase-3 (Cat. #9664), phosphoglycerate dehydrogenase (PHGDH) (Cat. #66,350), serine hydroxymethyltransferase 2 (SHMT2) (Cat. #33,443), glyceraldehyde-3-phosphate dehydrogenase (GAPDH) (Cat. #5568), and β-Tubulin (Cat. #5568) were purchased from Cell Signaling Technology (Danvers, MA, USA). Antibodies against DDDDK tag (Cat. Ab205606), Ki67 (Cat. Ab15580), and forkhead box K1 (FOXK1) (Cat. Ab309510) were purchased from Abcam (Cambridge, UK). The abovementioned antibodies were used at a 1:1000 dilution for western blotting. Additionally, the anti-ZSWIM4 antibody (Cat. PA5-59,861, Thermo Fisher Scientific, Waltham, MA, USA) was used at a 1:50 dilution for immunohistochemistry (IHC), and the anti-ZSWIM4/ cyanine3 (Cy3) antibody (Cat. Orb973969, Cambridge, UK) was used at a 1:200 dilution for flow cytometry and immunofluorescence. Chemical compounds (CBP, glycine, hypoxanthine, GSH, mirabegron, nelociguat, and olaparib) were purchased from MedChemExpression (Shanghai, China) and IPN60090 was obtained from TargetMol (Shanghai, China). Short hairpin (sh) ribonucleic acid (RNA) (shRNA) against *ZSWIM4* and lentivirus plasmids expressing *FOXK1* were purchased from Genecopoeia (Guangzhou, China). Wild-type and mutant *ZSWIM4* promoter-luciferase reporter plasmids were constructed from Genecopoeia by cloning the 1500 bp fragment before the *ZSWIM4* gene transcriptional start site into the pEZX-PL01 plasmid. Small interfering RNA against *ZSWIM4* and *SHMT2* and lentivirus plasmids expressing *ZSWIM4* were purchased from IGE Biotechnology (Guangzhou, China). Single guide RNA against *FOXK1* was packaged as lentiviruses (Genechem, Shanghai, China). The sequences mentioned above are presented in Additional file 1: Table S1.

### Cell viability assay

For the cell viability assay, EOC cells were cultured at a density of 5,000 cells/well in 96-well plates and treated with test compounds for the indicated time points. In the experiment using CBP, physiological saline was used as the control treatment. The cell viability was evaluated using a Cell Counting Kit-8 (CCK-8) (Cat. KGA317, KeyGen, Nanjing, China). Construction and statistical analysis of the drug 50% maximal inhibitory concentration (IC_50_) curves were carried out using GraphPad Prism ver. 6.0. as previously described [14]. Statistical analyses (mean ± standard deviation (SD)) of triplicates are presented.

### Western blot analysis

Cells were lysed in RIPA Lysis Buffer (Cat. KGP703, KeyGen) plus 1% phenylmethanesulfonyl fluoride (PMSF; Cat. KGP610, KeyGen). Protein concentration was detected using a BCA kit (Cat. KGP902, KeyGen). The proteins were separated by SDS-PAGE and then transferred onto a polyvinylidene fluoride membrane (Cat. ISEQ00010, Millipore, Bedford, MA, USA). The membranes were blocked and incubated with primary antibodies overnight at 4 °C and subsequently incubated with an appropriate secondary antibody (1:10,000) for 1 h. Protein bands were detected using ECL reagents (Cat. KGP1121, KeyGen).

### IHC, hematoxylin–eosin (H&E) staining, and immunofluorescence analysis

IHC analysis was performed using an IHC kit (Cat. IHC001, Bioss, Beijing, China). Briefly, after deparaffinization and rehydration, the slides were boiled in 0.01 M, pH 8.0 ethylenediaminetetraacetic acid for antigen retrieval. Subsequently, the slides were incubated in 3% H_2_O_2_ for 30 min to block endogenous peroxidase activity. Next, the slides were incubated with primary antibodies for 2 h at 37 °C, followed by treatment with a secondary antibody and diaminobenzidine. Published standards were referred to establish cutoffs for “low” and “high” ZSWIM4 expression and Ki67 scores [24]. For H&E staining, the sections were stained in hematoxylin for 5 min and eosin for 5 min. For the immunofluorescence assay, cells were fixed, permeabilized, and blocked, followed by incubation with primary antibodies overnight at 4 °C. Cells were then washed, incubated with secondary antibodies, and stained with DAPI (Cat. D-9106, Bioss).

### Quantitative reverse transcription polymerase chain reaction (qRT-PCR) assay

*AG RNAex Pro RNA* reagent (Cat. AG21101), *Evo M-MLV* RT Kit with gDNA Clean for qPCR (Cat. AG11705), and SYBR Green Premix *Pro Taq* HS qPCR (Cat. AG11701) were obtained from Accurate Biotechnology (ChangSha, China). RNA extraction and cDNA synthesis were performed following the manufacturer’s protocol. The resulting cDNA was then amplified on the Biosystems 7500 Real-time PCR system. The primers are listed in Additional file 1: Table S2.

### Flow cytometry

The Annexin V-FITC reagent (Cat. KGF001, KeyGen) and the Annexin V-APC apoptosis detection kit (Cat. KGA1021, KeyGen) were used to detect apoptosis. A reactive oxygen species (ROS) detection kit (Cat. S0033S, Beyotime, Shanghai, China) was used to evaluate ROS levels according to the manufacturer's protocol. The anti-ZSWIM4/Cy3 antibody was used to detect ZSWIM4 protein expression levels in *ZSWIM4-*knockdown clones. All flow cytometry results were analyzed using the FlowJo software (Tree Star Inc., USA).

### Tumorsphere assay

Briefly, 1 × 10^4^ EOC cells were resuspended and plated on the ultra-low attachment six-well plates. The tumorspheres were cultured in Dulbecco’s Modified Eagle Medium (DMEM)/F-12 (Gibco) with epidermal growth factor, insulin, hydrocortisone, and B27 supplement. Cells were treated with 50 μM CBP for 5 days, and tumorspheres with a diameter > 100 μm were counted.

### RNA-sequencing (RNA-seq) data analysis

Total RNA was isolated from *ZSWIM4*-knockdown and vector control OVCAR8 cells. A minimum of 5 μg total RNA was used for RNA-seq. RNA-seq and raw data analysis were performed by Novogene (Beijing, China). Gene Set Enrichment Analysis (GSEA) was performed for gene functional annotation and pathway analysis and the detailed results are presented in Additional file 1: Table S3.

### Luciferase reporter assay

Briefly, cells were transfected with the reporter plasmids using Lipofectamine™ 3000 (Cat. L3000015, Thermo Fisher Scientific). Luciferase activity was determined using a Microplate Reader (BioTek, Vermont, USA). *ZSWIM4* gene luciferase reporter activities were normalized to that of the renilla luciferase.

### CUT&Tag

For the CUT&Tag assay, a CUT&Tag-IT™ Assay Kit, Anti-Rabbit (Cat. 53,160, Active Motif, Shanghai, China) was used following the manufacturer’s instructions. Briefly, OVCAR8 and SKOV3 cells were harvested and washed with pre-chilled PBS. FOXK1 antibody mixture was added to the cell lysate and incubated for 2 h at 25 °C. Subsequently, the cell lysate was further processed for 30 min incubation with a secondary antibody. After centrifugation, the Dig Buffer and CUT&Tag-Tn5 enzyme mixture was added and incubated at 25 °C for 1 h. Next, the products were purified, and the PCR cycle number was determined. To perform CUT&Tag sequencing, the Illumina NovaSeq 6000 sequencing platform was employed in the PE150 sequencing mode. The recommended sequencing volume gene was 6G, and additional sequencing volume was performed according to the actual requirements. CUT&Tag sequencing data processing was performed by 10 K Genomics, Shanghai, China. The IGV tool was used to visualize the read count data.

### Chromatin immunoprecipitation (ChIP)

Approximately 5 × 10^6^ OVCAR8 and SKOV3 cells were fixed with cross-link solution and collected. ChIP assays were performed using a ChIP Kit (Cat. abs50034, Absin, Shanghai, China) according to the manufacturer’s instructions. Antibody-immunoprecipitated DNA was analyzed via real time PCR and normalized to the input.

### Assessment of intracellular glycine content

A Glycine Assay Kit (Fluorometric; Cat. ab211100, Abcam) was used to detect cellular glycine concentrations. Briefly, the collected cells were counted and further lysed. After being deproteinized using 10 kDa spin columns, the filtrate was collected to determine intracellular glycine content.

### Surface plasmon resonance (SPR) analysis

SPR analysis was used to detect the binding between the recombinant ZSWIM4 protein (Cat. TSTG2502, TargetMol) and IPN60090, with the ZSWIM4 protein and anti-ZSWIM4 antibody combination used as the positive control. The anti-ZSWIM4 antibody and IPN60090 were diluted to different concentrations using the running buffer (5% DMSO). The ZSWIM4 protein was coupled to the CM5 sensor chip (GE Healthcare, Marlborough, MA, USA) and served as the ligand. SPR experiments were performed using a BIAcore T200 instrument (GE Healthcare) at 25 °C following the manufacturer’s instruction. The BIAcore T200 Control software (v. 2.0, GE Healthcare) was used to analyze the binding model.

### Bioinformatics analysis

Differential expression analysis for *ZSWIM4* in ovarian tumors and control samples was performed using the Gene Expression Profiling Interactive Analysis (GEPIA) database [25]. The survival analysis based on *ZSWIM4* expression in OC was implemented using the GEPIA database and Kaplan–Meier plotter [26]. The gene expression levels of *ZSWIM4* and *FOXK1* in The Cancer Genome Atlas database (TCGA) and Etemadmoghadam 2009 were obtained from the UCSC Xena project [27], and the expression of *ZSWIM4* and *FOXK1* in platinum-resistant OVCAR8 cells were obtained from GSE45553 [28]. Virtual screening of ZSWIM4 targeted inhibitors was performed using Schrödinger Maestro ver. 11.4, and molecular docking between ZSWIM4 and IPN60090 was conducted using PyMol.

### Patient-derived organoid (PDO) model construction and culture

Fresh tumor tissues obtained from EOC patients were immersed in Advanced DMEM/F12 (Cat. 12634028, Thermo Fisher Scientific) containing 1% penicillin–streptomycin (PS). The necrotic tissues were removed, and the remaining tissue was cut into pieces of approximately 1–2 mm^3^ volume. After washing twice with PBS containing 1% PS, the tissue fragments were digested at 37 °C for 1 h using the Advanced DMEM/F12 solution containing type IV Collagenase (Cat. 17104019, Thermo Fisher Scientific). The above solution was filtered using a 100 μm filter (Cat. 352360, Corning, New York, USA). Next, the fragments were centrifuged at 300×*g* for 5 min. The erythrocytes were lysed. The precipitated cells were resuspended in the Advanced DMEM/F12 mixed with growth factor-reduced Matrigel (Cat. CB-40230, Corning, USA). The cell suspension was adjusted to a final concentration of 10,000–20,000 cells/50 μl. PDOs were transferred to 24-well plates when the Matrigel solidified, and 500 μl of the general culture medium was added. PDOs were grown in a cell incubator at 37 °C with 5% CO_2_. The culture medium was changed every 2–3 days. The cell viability of PDOs was determined by the Cell Counting-Lite 3D Luminescent Cell Viability Assay (Cat. DD1102-01, Vazyme, Nanjing, China).

### Animal experiments

Female BALB/c nude mice (4–6 weeks) were purchased from Guangdong Weitong Lihua Experimental Animal Technology. To evaluate the CBP sensitization effect of *ZSWIM4* knockdown in EOC cells in vivo, 1 × 10^7^ SKOV3 shCTR cells or *ZSWIM4-*knockdown cells were injected into the armpit of nude mice (*n* = 8/group). Once the tumor volume grew to 100  mm^3^, mice were administered CBP (20 mg/kg, i.p.) thrice weekly. After 2 weeks of treatment, the mice were sacrificed. To determine the efficacy of CBP combined with IPN60090 in vivo, 1 × 10^7^ SKOV3 cells were injected subcutaneously. When tumor volume reached 100  mm^3^, mice were treated with vehicle control, CBP (15 mg/kg, i.p.) thrice weekly, IPN60090 (30 mg/kg/day, p.o.), and CBP (15 mg/kg, i.p.) 3 weekly plus IPN60090 (30 mg/kg/day, p.o.) (*n* = 4/group). After 10 days of treatment, the mice were euthanized.

### Statistical analysis

Excluding the ZSWIM4 IHC results, which were expressed as median with interquartile range, all other data were expressed as mean ± SD and were analyzed using GraphPad Prism (6.0) software. The two groups were compared to obtain significant differences using Student’s *t*-test (two-tailed) or Welch's test for data with unequal variance. A one-way analysis of variance (ANOVA) was used to compare multiple groups, and a Mann–Whitney U-test was performed for IHC score data. A two-way ANOVA was performed to assess significant differences in tumor volume growth curves. The Fisher's exact test was adopted to evaluate differences in relative ZSWIM4 expression. The Gehan-Reslow-Wilcoxon test was used to determine the significance of survival data. Pearson’s correlation was used to determine the correlation between *ZSWIM4* and *FOXK1* expression. *P* values < 0.05 (*P < 0.05, **P < 0.01, ***P < 0.001) were considered significant, otherwise non-significant (ns).

## Results

### ZSWIM4 is highly expressed in EOC and is an indicator of poor prognosis in patients

**Fig. 1:**
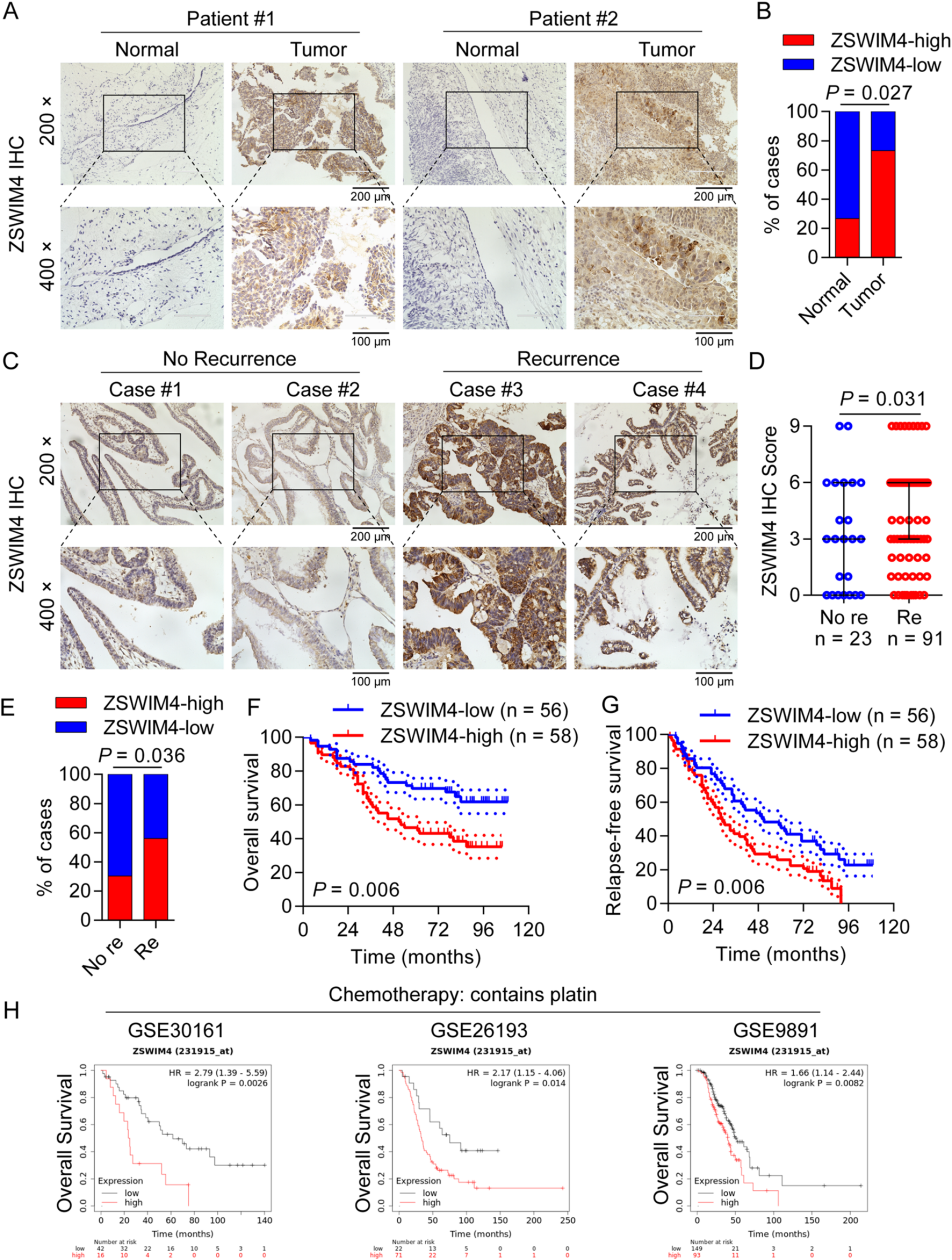
ZSWIM4 is highly expressed in epithelial ovarian cancer (EOC) and indicates poor prognosis. **A** IHC staining for ZSWIM4 in EOC tumor tissues and adjacent tissues; representative graphs are presented. **B** Statistical analysis of ZSWIM4 IHC score in EOC tumor tissues (*n* = 15) and adjacent tissues (*n* = 15). **C** IHC staining of ZSWIM4 in primary tumor tissues from patients with recurrent and non-recurrent EOC; representative graphs are presented. **D** IHC scores of ZSWIM4 in the recurrent and non-recurrent groups. Re, recurrence. **E** Statistical analysis of ZSWIM4 protein expression in primary tumor tissues from patients with recurrent (*n* = 91) and non-recurrent (*n* = 23) EOC. Re, recurrence. **F**, **G** Survival analysis of 114 EOC patients based on ZSWIM4 expression in TMA. Overall survival **F**. Relapse-free survival **G**. **H** Survival analysis of *ZSWIM4* expression in OC patients treated with platinum-based drugs using the Kaplan–Meier plotter website

First, by analyzing the data from the GEPIA database, we found that *ZSWIM4* was overexpressed in OC tumor tissues (Additional file 1: Fig. S1A), and that poor prognosis was associated with high *ZSWIM4* expression (Additional file 1: Fig. S1B).We consistently confirmed poorer prognosis in patients with OC showing *ZSWIM4* overexpression using the Kaplan–Meier plotter website (Additional file 1: Fig. S1C, D). Subsequently, we performed IHC to verify the overexpression of ZSWIM4 protein using EOC tumor tissues (*n* = 15) and control tissue samples (*n* = 15), including 10 paired samples. Interestingly, 11 of the 15 cancer tissues showed high ZSWIM4 expression, whereas only four of the 15 normal control tissues showed ZSWIM4 expression, indicating the upregulation of ZSWIM4 protein levels in EOC (Fig. 1A, B). Subsequently, we performed an IHC analysis for ZSWIM4 using an OC TMA. Based on the staining intensity and pattern, 114 valid ZSWIM4 expression scores were collected. In those 114 EOC tumors, the expression of ZSWIM4 among different EOC tissue types showed no significant difference (Additional file 1: Fig. S1E). Notably, we found a significant increase of ZSWIM4 expression in relapsed primary cancers (Fig. 1C, D) and a positive correlation between high ZSWIM4 expression and primary tumor recurrence based on the clinical information (Fig. 1E). Survival analysis results based on the TMA also revealed that high expression of ZSWIM4 could predict poorer overall survival rates (Fig. 1F) and higher postoperative relapse rates (Fig. 1G) in patients with EOC. Furthermore, to investigate the factors contributing to the effects of ZSWIM4 on EOC recurrence, we evaluated its prognostic role in patients with this disease treated with platinum-based chemotherapy using the Kaplan–Meier plotter database. Notably, high *ZSWIM4* expression in OC indicated a poor prognosis for the patients administered platinum-based treatment (Fig. 1H), suggesting that high ZSWIM4 expression in EOC tumor tissues might counter the effect of platinum drugs.

### Elevated ZSWIM4 expression reduces EOC cell sensitivity to CBP

**Fig. 2:**
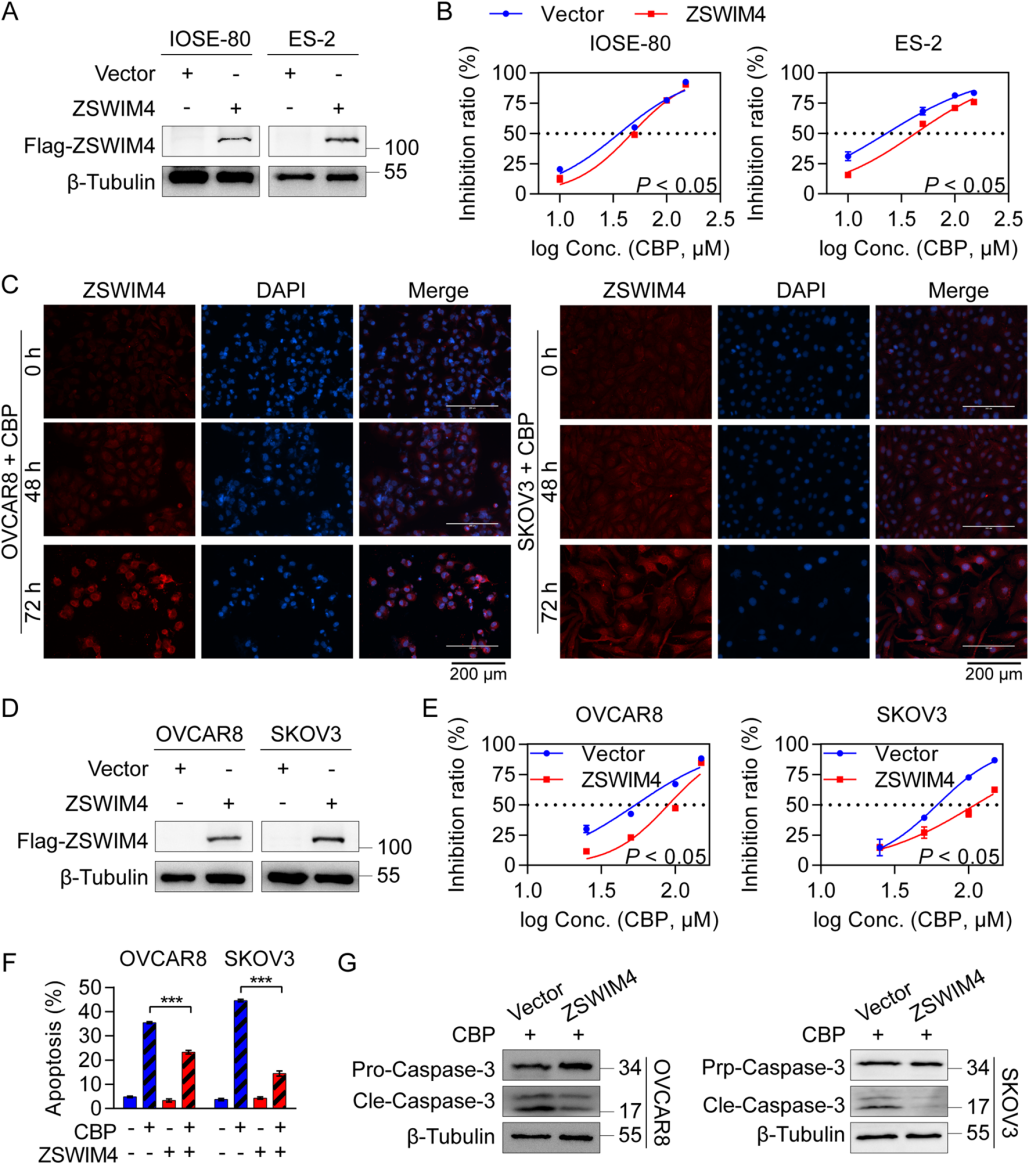
Elevated ZSWIM4 resists chemotherapy in epithelial ovarian cancer (EOC) cells. **A** ZSWIM4 protein expression levels in the vector control and ZSWIM4-overexpressing IOSE-80 and ES-2 cells. **B** IC_50_ curves for vector control and ZSWIM4-overexpressing IOSE-80 and ES-2 cells following incubation with a gradient concentration CBP for 72 h. **C** Immunofluorescence detection of ZSWIM4 protein expression in OVCAR8 and SKOV3 cells after CBP (75 μM) treatment for 48 or 72 h. **D** ZSWIM4 protein expression in the vector control and ZSWIM4-overexpressing OVCAR8 and SKOV3 cells. **E** IC_50_ curves for vector control and ZSWIM4-overexpressing OVCAR8 and SKOV3 cells following incubation with CBP for 72 h. **F** Statistical data (mean ± SD) of the apoptosis rate in ZSWIM4-overexpressing and vector control OVCAR8 and SKOV3 cells treated with the vehicle control or CBP (50 μM) treatment for 72 h. **G** Cleaved caspase-3 protein levels in ZSWIM4-overexpressing OVCAR8 and SKOV3 cells treated with CBP (50 μM) for 72 h detected via western blotting

To investigate the effect of ZSWIM4 in driving chemotherapy resistance, we selected two cell lines with low expression of this protein, IOSE-80 (human normal ovarian epithelial cells) and ES-2 (human ovarian clear cell carcinoma cells) cells (Additional file 1: Fig. S2A), to establish the ZSWIM4 overexpression models (Fig. 2A, Additional file 1: Fig. S2B). Subsequently, we treated both vector control and ZSWIM4-overexpressing clones with 50 μM CBP for 72 h, and observed that overexpression of ZSWIM4 conferred resistance to CBP in IOSE-80 and ES-2 cell lines (Additional file 1: Fig. S2C). In addition, exogenous ZSWIM4 expression increased the IC_50_ values of CBP in both IOSE-80 and ES-2 cell lines (Fig. 2B). In our previous study, we found that ZSWIM4 was an inducible drug resistance gene in breast cancer cells [14]. Notably, the messenger RNA (mRNA) (Additional file 1: Fig. S2D) and protein expression levels (Fig. 2C) of ZSWIM4 were significantly induced upon 75 μM CBP treatment in OVCAR8 and SKOV3 cells. To discern the pathological significance of inducible ZSWIM4 in EOC cells upon CBP treatment, stable clones of ZSWIM4-overexpressing OVCAR8 and SKOV3 cells were generated to mimic the above situations (Fig. 2D, Additional file 1: Fig. S2E). Then, both vector control and ZSWIM4-overexpressing EOC cells were incubated with 100 μM CBP for 72 h. We found that the up-regulation of ZSWIM4 compromised the cytotoxic effect of CBP, as indicated by the CCK-8 assay results (Additional file 1: Fig. S2F). Furthermore, we generated IC_50_ curves and found that ZSWIM4 overexpression increased the IC_50_ values of CBP (Fig. 2E). Moreover, the overexpression of ZSWIM4 weakened CBP-mediated suppression of tumorsphere formation in OVCAR8 and SKOV3 cells (Additional file 1: Fig. S2G). Subsequently, we characterized CBP-induced cell apoptosis in vector control and ZSWIM4-overexpressing OVCAR8 and SKOV3 cells incubated with 50 μM CBP for 72 h. Overexpression of ZSWIM4 significantly ameliorated CBP-induced apoptosis, as evidenced by the reduced Annexin V-positive cell proportions (Fig. 2F, Additional file 1: Fig. S2H) and the downregulation of cleaved caspase-3 (Fig. 2G). These data suggest that high ZSWIM4 expression in EOC cells could reduce their sensitivity to CBP by inhibiting CBP-mediated apoptosis.

### Silencing ZSWIM4 enhances EOC cell sensitivity to CBP

**Fig. 3:**
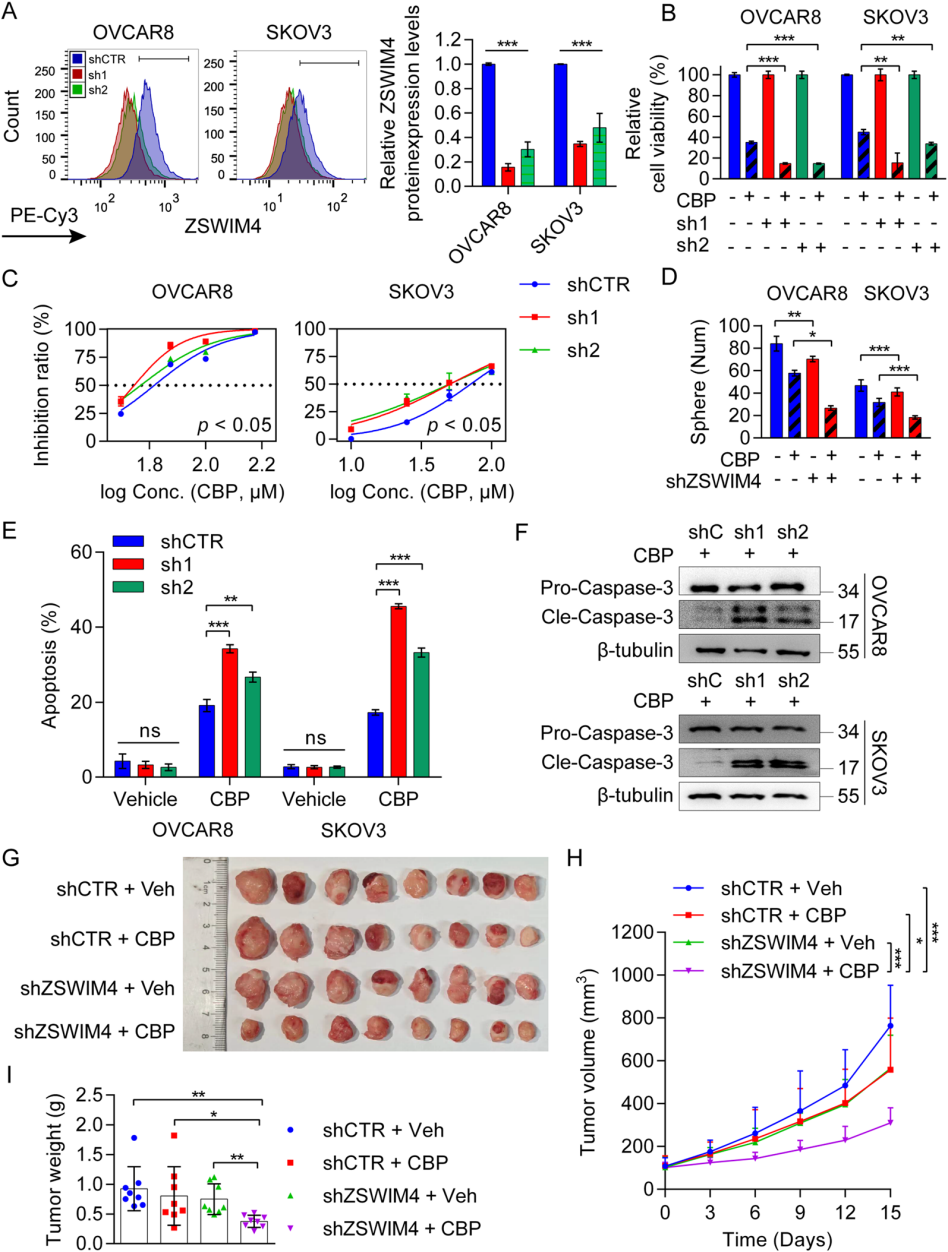
Silencing ZSWIM4 enhances the sensitivity of epithelial ovarian cancer (EOC) cells to CBP. **A** ZSWIM4 protein expression levels in the vector control and shRNA-mediated *ZSWIM4*-knockdown EOC clones. Representative flow cytometry histograms (*left*) and statistical data (mean ± SD) are shown (*right*). **B** Killing effects in *ZSWIM4*-knockdown and shRNA control OVCAR8 cells treated with CBP (100 μM) for 72 h and *ZSWIM4*-knockdown and shRNA control SKOV3 cells treated with CBP (50 μM) for 72 h; CCK-8 assays. **C** CBP sensitivity of *ZSWIM4*-knockdown OVCAR8 and SKOV3 cells. **D** The statistical data (mean ± SD) of tumorspheres of control and *ZSWIM4*-knockdown EOC cells incubated in 50 μM CBP for 5 days. **E** The statistical data (mean ± SD) of apoptosis rate of *ZSWIM4*-knockdown and control EOC cells with or without CBP (50 μM) treatment for 72 h. **F** The protein expression levels of cleaved caspase-3 in CBP-treated *ZSWIM4*-knockdown EOC cells. shC, shCTR. **G**–**I** The shRNA control and *ZSWIM4*-knockdown SKOV3-derived xenograft mice were treated with vehicle control or CBP (20 mg/kg thrice weekly) for two weeks (*n* = 8/group). Photographs of tumors **G**. Growth curves of xenograft tumors **H**. Tumor weight **I**. Veh, vehicle

To better clarify the impact of ZSWIM4 on chemosensitivity, we knocked down *ZSWIM4* in OVCAR8 and SKOV3 cells (Fig. 3A, Additional file 1: Fig. S3A). Subsequently, we treated *ZSWIM4*-knockdown OVCAR8 cells and control cells with 100 μM CBP and *ZSWIM4*-knockdown SKOV3 cells and control cells with 50 μM CBP for 72 h. Cell viability assays unveiled that *ZSWIM4*-knockdown EOC cells were more sensitive to CBP treatment (Fig. 3B). Furthermore, after incubation with a gradient concentration of CBP for 72 h, the IC_50_ values declined in both *ZSWIM4*-knockdown EOC cells (Fig. 3C), indicating that *ZSWIM4* knockdown enhances EOC cell sensitivity to chemotherapy. *ZSWIM4* silencing facilitated the CBP-mediated inhibition of tumorsphere formation (Fig. 3D, Additional file 1: Fig. S3B) and enhanced CBP-mediated apoptosis (Fig. 3E, Additional file 1: Fig. S3C). Consistently, the increased expression of cleaved caspase-3 in the CBP-treated *ZSWIM4*-knockdown EOC clones validated the above results (Fig. 3F). Furthermore, we analyzed the response of vector control cells and *ZSWIM4*-knockdown cells to CBP in vivo. For this, we injected the *ZSWIM4*-knockdown or control SKOV3 cells into nude mice. Then, CBP treatment at a dose of 20 mg/kg three times per week was initiated when xenograft tumor volume reached approximately 100  mm^3^. After 2 weeks of treatment, we found that the knockdown of *ZSWIM4* also sensitized EOC cells to CBP in vivo (Fig. 3G), as indicated by the slower growth curve (Fig. 3H) and reduced tumor weights (Fig. 3I) of subcutaneous tumors among the four groups of mice. The decreased Ki67 proliferation score also confirmed these results (Additional file 1: Fig. S3D). These data revealed that ZSWIM4 inhibition improves chemosensitivity in EOC cells and tumors.

### FOXK1-dependent ZSWIM4 transcription reduces EOC cell sensitivity to CBP

**Fig. 4:**
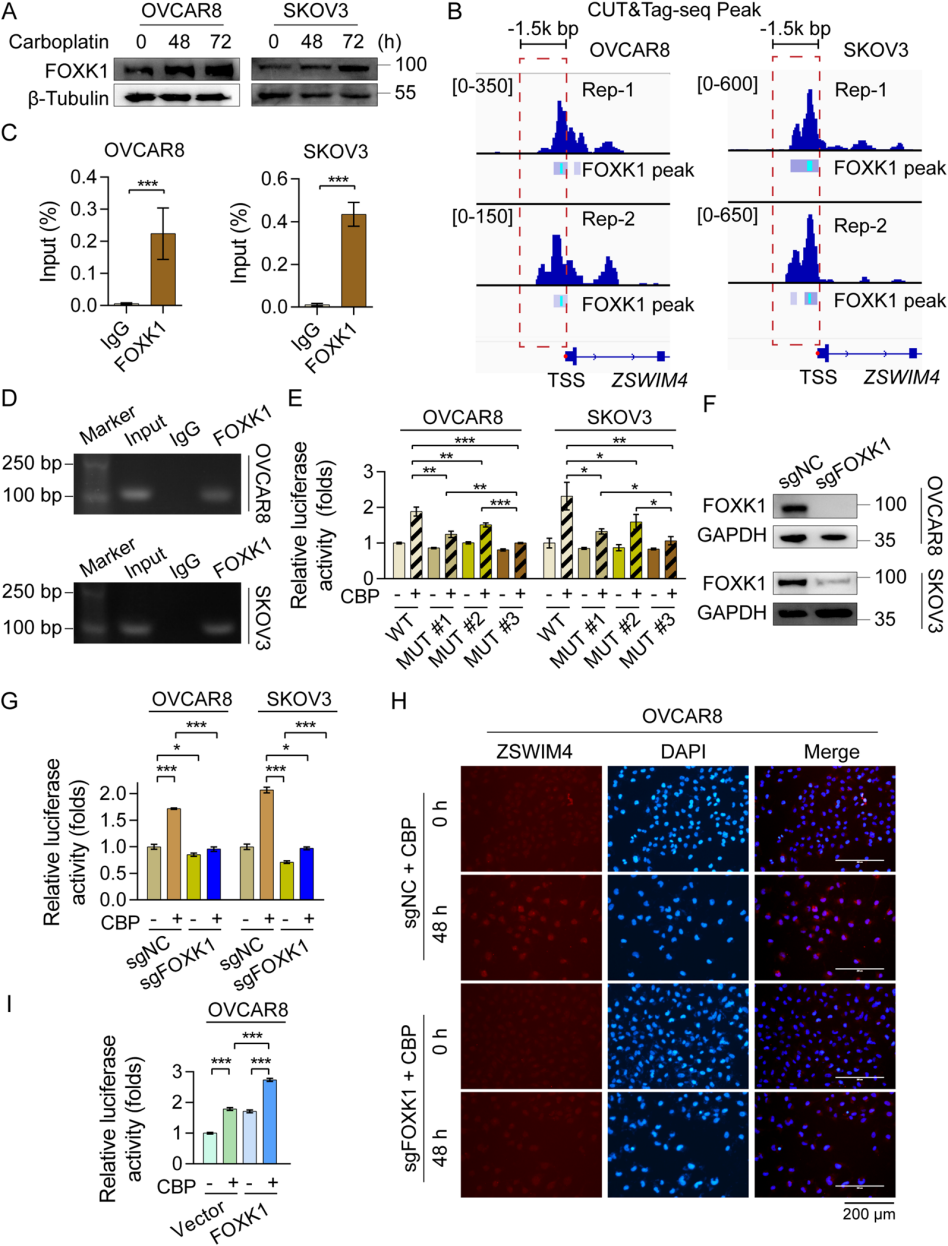
FOXK1-dependent ZSWIM4 transcription contributes to epithelial ovarian cancer (EOC) cell chemotherapy resistance. **A** FOXK1 protein levels in both EOC cell lines treated with vehicle or CBP (75 μM) for 0, 48, and 72 h. **B** CUT&Tag-seq analyses of FOXK1 binding peaks on the promoter regions of *ZSWIM4* in OVCAR8 and SKOV3 cells. Rep-1, replicated sample-1, Rep-2, replicated sample-2. **C** Enrichment of *ZSWIM4* promoter fragments using an antibody against FOXK1 was assessed by ChIP-qPCR analysis. **D** Agarose electrophoresis analysis of PCR products of the *ZSWIM4* promoter after ChIP-PCR assays. **E** *ZSWIM4* promoter activity in EOC cells transfected with wild-type or mutant plasmids in the presence or absence of 100 μM CBP for 24 h. **F** *FOXK1* knocked down in OVCAR8 and SKOV3 cells. **G** *ZSWIM4* promoter activity in FOXK1 silencing EOC cells treated with vehicle or CBP (75 μM) for 48 h. **H** Immunofluorescence analysis of ZSWIM4 expression in control and *FOXK1*-knockdown OVCAR8 cells treated with CBP (75 μM) for 48 h. **I** *ZSWIM4* promoter activity in OVCAR8 cells transfected with wild-type *ZSWIM4* promoter-luciferase plasmid with or without FOXK1 co-transfection. The cells were treated with vehicle or CBP (75 μM) for 48 h

We explored the mechanism underlying the induction of *ZSWIM4* expression upon CBP treatment. A previous study showed that the transcription factor FOXK1 is upregulated in platinum drug-resistant cell lines [29]. Based on public database analysis, strong positive correlations between *FOXK1* and *ZSWIM4* mRNA levels were observed in OC tumor tissues (Additional file 1: Fig. S4A). By analyzing gene expression in OVCAR8 parental cells and cisplatin-resistant cells, we observed co-upregulation of *FOXK1* and *ZSWIM4* in cisplatin-resistant OVCAR8 cells (Additional file 1: Fig. S4B). Our western blotting results revealed that FOXK1 abundance gradually increased upon CBP (75  μM) treatment within 72 h (Fig. 4A). By searching the promoter region of *ZSWIM4* in the JASPAR database, setting a filtering criterion of a score greater than 10 and a correlation greater than 0.9, we identified two potential FOXK1-binding sites (Additional file 1: Fig. S4C)*.* Next, we performed CUT&Tag-seq using an antibody against FOXK1 (Additional file 1: Fig. S4D) and found the common binding peak of FOXK1 in the promoter region of *ZSWIM4* loci in both OVCAR8 and SKOV3 cells (Fig. 4B). The ChIP assays were then conducted, and the results confirmed that FOXK1 bound directly to the *ZSWIM4* promoter region (Fig. 4C, D). Therefore, we hypothesized that *ZSWIM4* upregulation might be mediated by the transcriptional effect of FOXK1. To test our hypothesis, we constructed a wild-type *ZSWIM4* promoter-luciferase reporter vector by inserting the 1500 bp promoter fragment upstream of the *ZSWIM4* gene translation start site and three mutated *ZSWIM4* promoter-luciferase reporter vectors in which the FOXK1-binding sites were mutated (MUT #1, MUT #2, and MUT #3) (Additional file 1: Fig. S4E). As expected, 100  μM CBP treatment for 24  h enhanced *ZSWIM4* gene transcriptional activity in both OVCAR8 and SKOV3 cells, whereas the mutation in the FOXK1-recognition motif eliminated CBP-induced luciferase activity. Notably, mutations in two binding sites had a more significant effect on reducing the CBP-induced upregulation of *ZSWIM4* transcriptional activity than mutations in any single binding site (Fig. 4E). Furthermore, we knocked down *FOXK1* in OVCAR8 and SKOV3 cells to elucidate its role in inducing ZSWIM4 (Fig. 4F). Notably, the knockdown of *FOXK1* downregulated *ZSWIM4* (Additional file 1: Fig. S4F) and its transcriptional activity (Additional file 1: Fig. S4G). Simultaneously, silencing *FOXK1* reduced the upregulation of *ZSWIM4* promoter-luciferase activity (Fig. 4G) and inhibited the upregulation of ZSWIM4 induced by 75 μM CBP treatment for 48 h (Fig. 4H). We also generated stable FOXK1-overexpressing clones in OVCAR8 cells (Additional file 1: Fig. S4H). In line with our hypothesis, ectopic FOXK1 expression enhanced CBP-induced *ZSWIM4* promoter-luciferase activity (Fig. 4I). Moreover, knockdown of *ZSWIM4* in FOXK1-overexpressing OVCAR8 cells partially alleviated drug resistance to CBP (Additional file 1: Fig. S4I). Collectively, our results indicated that FOXK1-mediated *ZSWIM4* transcription was responsible for the decreased sensitivity of EOC cells to CBP.

### ZSWIM4 knockdown enhances CBP-induced reactive oxygen species (ROS) production

**Fig. 5:**
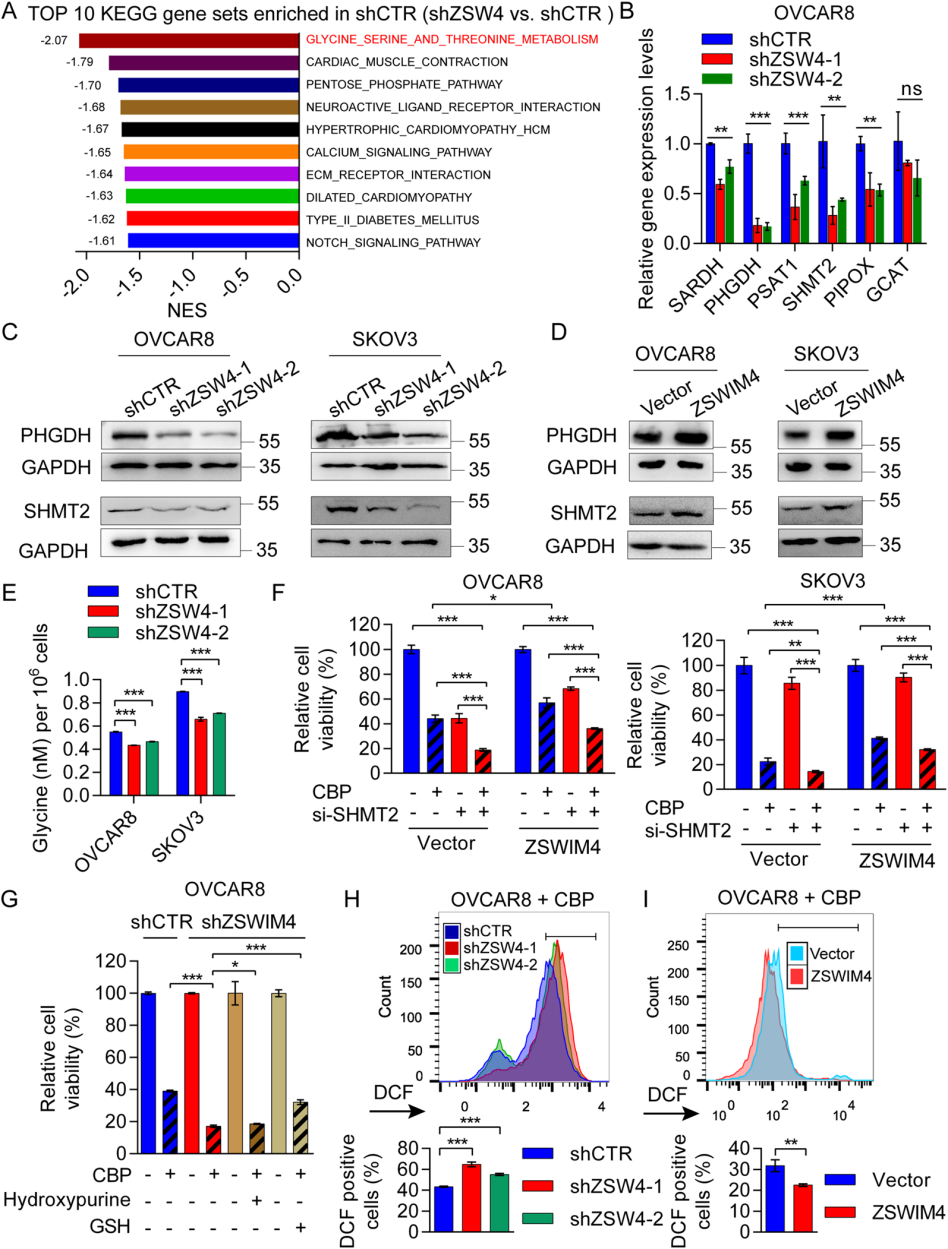
Glycine metabolism reprogramming is triggered after knocking down ZSWIM4 in EOC cells. **A** Kyoto Encyclopedia of Genes and Genomes (KEGG) pathway analysis in OVCAR8 cells after *ZSWIM4* knockdown. **B** The mRNA expression levels of genes encoding key enzymes for glycine synthesis in vector control and *ZSWIM4*-knockdown OVCAR8 cells. **C**, **D** Protein expression levels of PHGDH and SHMT2 in *ZSWIM4*-knockdown EOC clones **C** and ZSWIM4-overexpressing EOC clones **D**. **E** Intracellular glycine content of *ZSWIM4*-knockdown OVCAR8 and SKOV3 cells. **F** Viability of cells following *SHMT2* knockdown in vector control and ZSWIM4-overexpressing EOC cells treated with vehicle or CBP (100 μM) for 72 h; CCK-8 assay. **G** Rescue experiments using exogenous supplementation of hypoxanthine and GSH in *ZSWIM4*-knockdown OVCAR8 cells. **H** ROS levels in vector control and *ZSWIM4*-knockdown OVCAR8 cells after CBP (50 μM) treatment for 72 h. Representative histograms (*upper*) and statistical data (mean ± SD) are shown (*lower*). **I** Flow cytometry detection of ROS levels in vector control and ZSWIM4-overexpressing OVCAR8 cells treated with vehicle or CBP (50 μM) for 72 h. Representative histograms (*upper*) and statistical data (mean ± SD) (*lower*) are shown

To elucidate how ZSWIM4 confers decreased sensitivity to CBP in EOC cells, we performed next-generation RNA-seq using *ZSWIM4*-knockdown and control OVCAR8 cells. Pathway enrichment analysis demonstrated that “GLYCINE_SERINE_AND_THREONINE_METABOLISM” was the most significantly downregulated pathway after *ZSWIM4* knockdown (Fig. 5A, Additional file 1: Fig. S5A). By investigating the members of this pathway, we found that most of the downregulated genes-encoded key enzymes participate in the synthesis of serine and glycine (Additional file 1: Fig. S5B). Among them, the expression levels of *PHGDH*, the gene encoding phosphoglycerate dehydrogenase, and *SHMT2*, the gene encoding serine hydroxymethyltransferase 2, were the lowest (Fig. 5B). In addition, we confirmed that *ZSWIM4* knockdown reduced the expression of PHGDH and SHMT2 (Fig. 5C), whereas the overexpression of ZSWIM4 upregulated PHGDH and SHMT2 expression in OVCAR8 and SKOV3 cells (Fig. 5D). PHGDH catalyzes intracellular serine biosynthesis [30]. Serine is further converted into glycine by SHMT, and SHMT2 is the rate-limiting enzyme in this step [31]. A previous study indicated that *PHGDH* knockdown enhances EOC cell chemosensitivity [32]. Notably, in both EOC cell lines, *ZSWIM4* knockdown decreased the intracellular glycine content (Fig. 5E), whereas exogenous glycine supplementation partially restored the sensitivity of OVCAR8 cells to CBP induced by *ZSWIM4* knockdown (Additional file 1: Fig. S5C). Therefore, we investigated whether the downregulation of SHMT2 after *ZSWIM4* knockdown could also enhance the chemotherapy sensitivity in EOC cells. For this, we knocked down *SHMT2* in OVCAR8 and SKOV3 cells and observed that silencing *SHMT2* enhanced the sensitivity of CBP treatment in both two EOC cell lines (Additional file 1: Fig. S5D). Consistently, 50 or 100 µM CBP in combination with the SHMT inhibitor (SHIN1) showed a stronger killing effect in both OVCAR8 and SKOV3 cells than CBP treatment alone (Additional file 1: Fig. S5E). Next, we knocked down *SHMT2* in ZSWIM4-overexpressing clones and treated control and *SHMT2*-knockdown cells with 100 µM CBP. *SHMT2* silencing partially rescued the reduced sensitivity of CBP caused by *ZSWIM4* overexpression in both EOC cell lines (Fig. 5F), indicating that SHMT2 is a downstream target of ZSWIM4. SHMT2 catalyzes the synthesis of glycine, which is involved in various metabolic pathways, such as purine nucleotide and GSH synthesis [33]. We treated control cells and *ZSWIM4*-knockdown OVCAR8 cells with 100 µM CBP and exogenously administered hypoxanthine (100 µM) and GSH (100 µM) to the knockdown group. Interestingly, GSH partially rescued the effects of *ZSWIM4* knockdown, whereas hypoxanthine did not (Fig. 5G). GSH is an important regulator of intracellular redox homeostasis [34]. Furthermore, we observed a CBP-induced increase in intracellular ROS levels following *ZSWIM4* knockdown (Fig. 5H) and decreased ROS levels in ZSWIM4-overexpressing OVCAR8 cells after CBP (50 μM) treatment (Fig. 5I). These results suggest that *ZSWIM4* knockdown downregulates the glycine synthesis enzymes, leading to a diminution in intracellular glycine content, and augments the CBP-induced ROS levels, thereby enhancing the effect of CBP on EOC cells.

### A ZSWIM4 inhibitor enhances the sensitivity of EOC cells to CBP

**Fig. 6:**
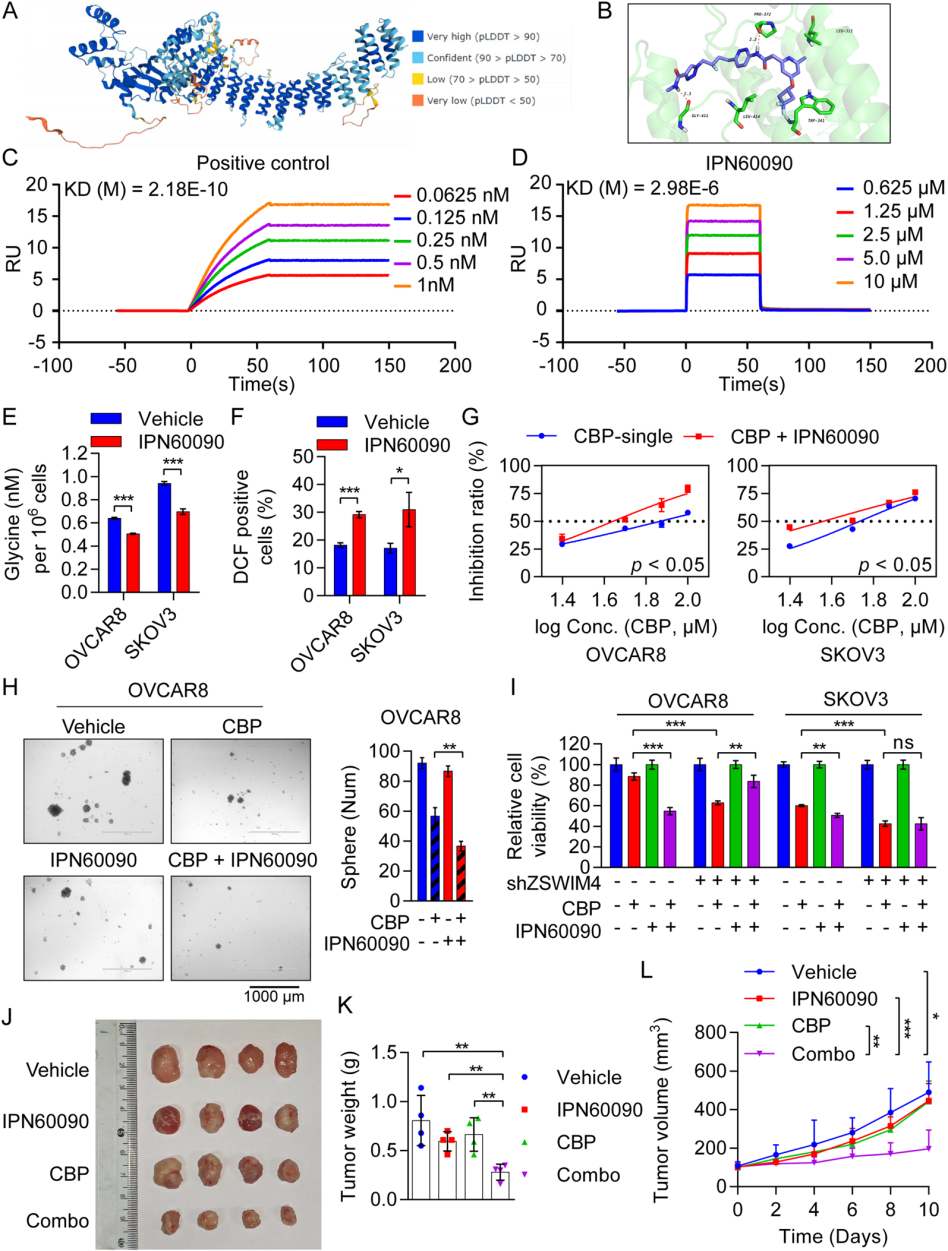
IPN60090, a ZSWIM4-targeting inhibitor, enhances the chemotherapeutic sensitivity of EOC cells. **A** 3D structure of human ZSWIM4. **B** Structural simulation of ZSWIM4 and IPN60090. IPN60090 is depicted as a gray-white stick, with the red dashed line indicating the length of the hydrogen bond. **C**–**D** The affinity of anti-ZSWIM4 antibody **C** and IPN60090 **D** for ZSWIM4 protein determined by SPR analyses. KD, dissociation constant. **E** Detection of intracellular glycine content in OVCAR8 and SKOV3 cells treated with 10 μM IPN60090 for 48 h. **F** ROS levels in EOC cells following IPN60090 (10 μM) treatment for 48 h. **G** IC_50_ curves for OVCAR8 and SKOV3 cells following treatment with CBP and/or IPN60090 (10 μM) for 72 h. **H** Tumorsphere formation in OVCAR8 cells treated with vehicle control, CBP (50 μM), IPN60090 (10 μM), and CBP (50 μM) combined with IPN60090 (10 μM) for 5 day. Representative images (*left*) and statistical data (mean ± SD) (*right*) are shown. **I** Viability of *ZSWIM4*-knockdown and vector control EOC cells following treatment with CBP (25 μM) with or without IPN60090 (10 μM) for 72 h; CCK-8 assay. **J**–**L** SKOV3-derived xenograft mice were divided into four groups (*n* = 4/group) and administered the vehicle control, CBP (15 mg/kg, i.p.) thrice weekly, IPN60090 (30 mg/kg/day, p.o.), and CBP combined with IPN60090. Photographs of tumors **J**. Tumor weight **K**. Growth curves of xenograft tumors **L**

Currently, there are few reports on ZSWIM4-targeting inhibitors. Therefore, we attempted to identify small-molecule inhibitors targeting this protein using computerized virtual screening. We predicted the three-dimensional (3D) structure of ZSWIM4 using AlphaFold, and a high percentage of 3D structures scored > 70 points (Fig. 6A), indicating that this prediction had high structural reliability. Next, we found that the Site1 binding pocket in the 3D structure had a strong ability to accept compounds (Additional file 1: Fig. S6A). Therefore, we performed a virtual screening against this Site1 locus using the MedChemExpress Bioactive Compound Library Plus and yielded three small-molecule compounds (mirabegron, IPN60090, and nelociguat) with a docking score of less than ˗10.5 (Additional file 1: Fig. S6B). Subsequently, we treated OVCAR8 cells with 25 μM CBP in combination with the three drugs (all at a concentration of 10 μM). Notably, only IPN60090 enhanced the sensitivity of OVCAR8 cells to CBP (Additional file 1: Fig. S6C). IPN60090 was initially developed as an inhibitor of glutaminase-1 (GLS-1), and it has entered clinical trials for cancer therapy, especially for lung and ovarian cancers [35, 36]. Therefore, we further investigated the interaction between IPN60090 and ZSWIM4. Docking results indicated that IPN60090 (Additional file 1: Fig. S6D) binds to the ZSWIM4 protein by forming hydrogen bonds with the residues proline 204 (Pro372) and glycine 411 (Gly411) in ZSWIM4 (Fig. 6B). Subsequently, using an anti-ZSWIM4 antibody as positive control (Fig. 6C), we performed an SPR assay to detect the affinity between the recombinant ZSWIM4 protein (Additional file 1: Fig. S6E) and IPN60090. The results showed that IPN60090 can specifically bind to the ZSWIM4 protein (Fig. 6D). Furthermore, IPN60090 was found to downregulate the intracellular glycine levels (Fig. 6E) and elevate ROS levels (Fig. 6F, Additional file 1: Fig. S6F) in OVCAR8 and SKOV3 cells. Furthermore, cell viability assays showed that 50 μM CBP in combination with 10 μM IPN60090 exhibited a stronger cytotoxic effect (Additional file 1: Fig. S6G), and IC_50_ curve analyses revealed that the ZSWIM4 inhibitor had a chemosensitizing effect (Additional file 1: Fig. 6G). In addition, the combination of IPN60090 and CBP inhibited the formation of tumorspheres in both OVCAR8 and SKOV3 cells (Fig. 6H, Fig. S6H). To clarify whether IPN60090 exerts CBP-sensitizing effects by inhibiting ZSWIM4, we treated both *ZSWIM4*-knockdown and control EOC cells with 25  μM CBP alone or 25  μM CBP combined with IPN60090 (10 μM) for 72 h. Interestingly, IPN60090 lost its synergistic effect with CBP in *ZSWIM4*-knockdown cells (Fig. 6I). Consistently, IC_50_ curve analyses suggested that IPN60090 could be used to overcome CBP resistance caused by ZSWIM4 overexpression in OVCAR8 cells (Additional file 1: Fig. S6I). Considering that poly (ADP-ribose) polymerase (PARP) inhibitors are also used for the clinical treatment of EOC [1] and metabolic reprogramming can cause EOC cells to resist PARP inhibitors [21], we attempted to clarify whether the use of ZSWIM4 inhibitors could also enhance the sensitivity to PARP inhibitors. We treated both OVCAR8 and SKOV3 cells with 50 or 100 µM olaparib in combination with IPN60090 (50 µM) for 48 h, and the results showed that this cotreatment resulted in a stronger killing effect than olaparib treatment alone (Additional file 1: Fig. S6J). Furthermore, we evaluated the chemotherapeutic sensitization effect of the ZSWIM4 inhibitor IPN60090 in vivo using SKOV3-derived xenograft mice*.* When the tumor volume reached 100 mm^3^, the mice were administered the vehicle control, CBP (15 mg/kg, i.p.) 3 weekly, IPN60090 (30 mg/kg/day, per os (p.o.)), and CBP combined with IPN60090. After 10 days of drug treatment, the mice were euthanized. IPN60090 significantly enhanced the inhibitory effect of CBP in vivo (Fig. 6J–L). Collectively, our data revealed that the use of a ZSWIM4 inhibitor exerts marked synergistic effects with CBP in killing EOC cells, like those with *ZSWIM4* knockdown.

### The ZSWIM4 inhibitor enhances CBP sensitivity in CBP-resistant EOC cells

**Fig. 7:**
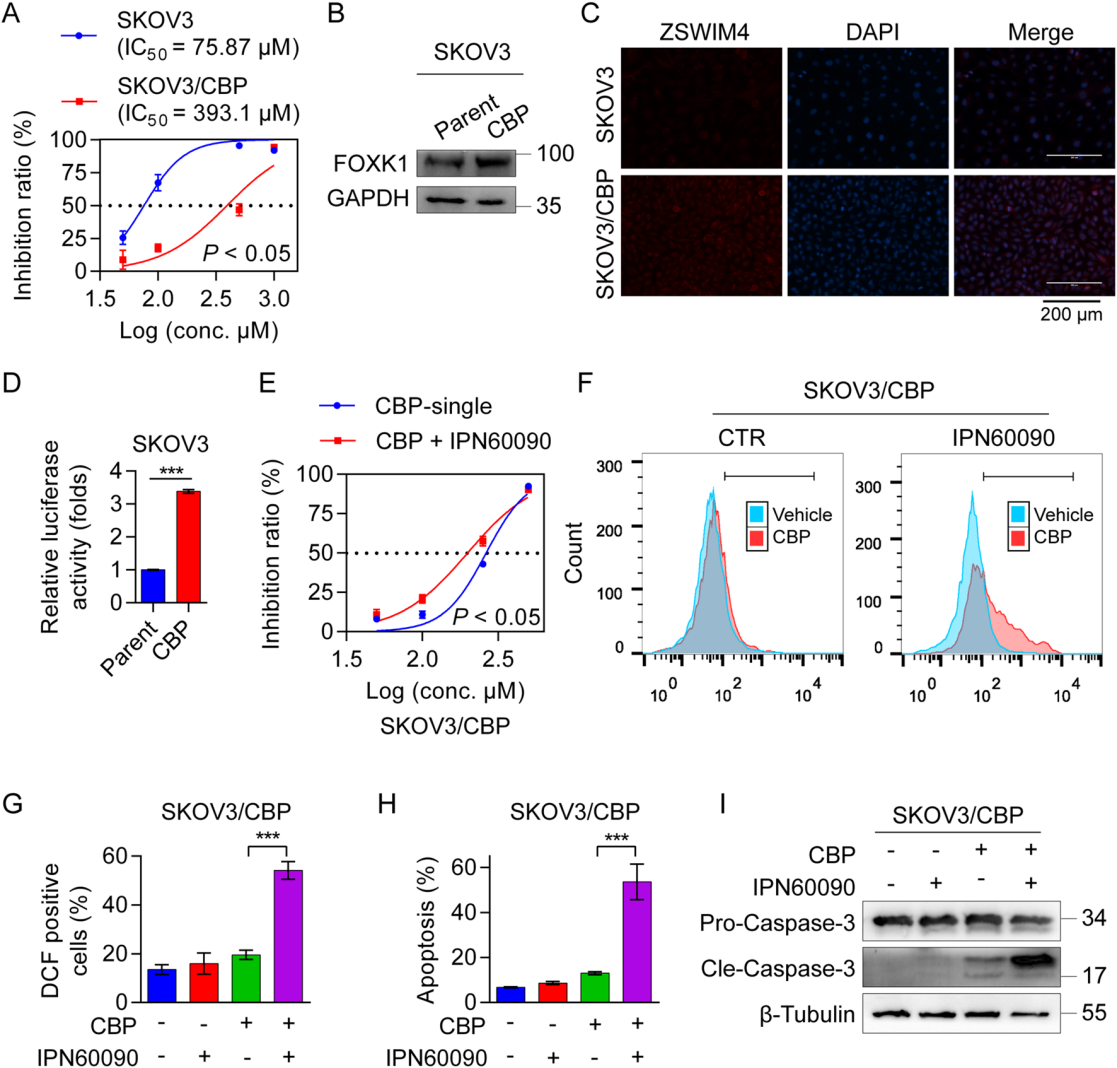
ZSWIM4 inhibitor enhances chemotherapy sensitivity in CBP-resistant SKOV3 cells. **A** Drug sensitivity of CBP in parental SKOV3 and SKOV3/CBP cells. **B** The protein expression of FOXK1 in parental SKOV3 and SKOV3/CBP cells. **C** Immunofluorescence detection of ZSWIM4 protein expression in parental SKOV3 and SKOV3/CBP cells. **D** *ZSWIM4* gene promoter-luciferase reporter activity in parental SKOV3 and SKOV3/CBP cells. **E** IC_50_ curves of SKOV3/CBP cells with a gradient concentration of CBP with or without IPN60090 (50 μM) treatment for 72 h. (**F**–**I**) SKOV3/CBP cells treated with vehicle, CBP (200 μM), IPN60090 (50 μM), and CBP (200 μM) combined with IPN60090 (50 μM) for 72 h. ROS levels **F**, and statistical data (mean ± SD) **G**. Flow cytometry analysis of cell apoptosis rate (mean ± SD) **H**. Cleaved caspase-3 protein abundance **I**

Furthermore, we established CBP-resistant SKOV3 clones (SKOV3/CBP) using parental SKOV3 cells by increasing the concentration of CBP stepwise. The IC_50_ value of CBP in SKOV3/CBP cells was more than five-fold higher than that in the parental SKOV3 cells (Fig. 7A). We observed higher FOXK1 protein abundance in SKOV3/CBP cells than in the parental cells (Fig. 7B). ZSWIM4 was consistently upregulated in the CBP-resistant clones (Fig. 7C). Thereafter, we transfected the *ZSWIM4* promoter-luciferase plasmid into both parental and CBP-resistant SKOV3 cells and obtained an elevated luciferase reporter activity in SKOV3/CBP cells (Fig. 7D). These data suggested that FOXK1/ZSWIM4 signaling was highly activated in SKOV3/CBP cells. Subsequently, we treated SKOV3/CBP cells with 200 μM CBP combined with or without 50 μM IPN60090. As expected, the co-treatment resulted in a better killing effect (Additional file 1: Fig. S7A). Moreover, IC_50_ curve analyses revealed that the addition of the ZSWIM4 inhibitor re-sensitized SKOV3/CBP cells to CBP (Fig. 7E). Flow cytometric results further confirmed that co-treatment increased intracellular ROS levels (Fig. 7F, G) and robustly enhanced apoptosis in SKOV3/CBP cells (Fig. 7H, Additional file 1: Fig. S7B). Western blotting results also revealed that CBP (200 μM) co-treated with IPN60090 (50 μM) induced markedly stronger expression of cleaved caspase-3 (Fig. 7I). Collectively, our data support the hypothesis that ZSWIM4 inhibition sensitizes EOC cells to chemotherapy.

### ZSWIM4 inhibition sensitizes EOC cells to chemotherapy in PDO models

**Fig. 8:**
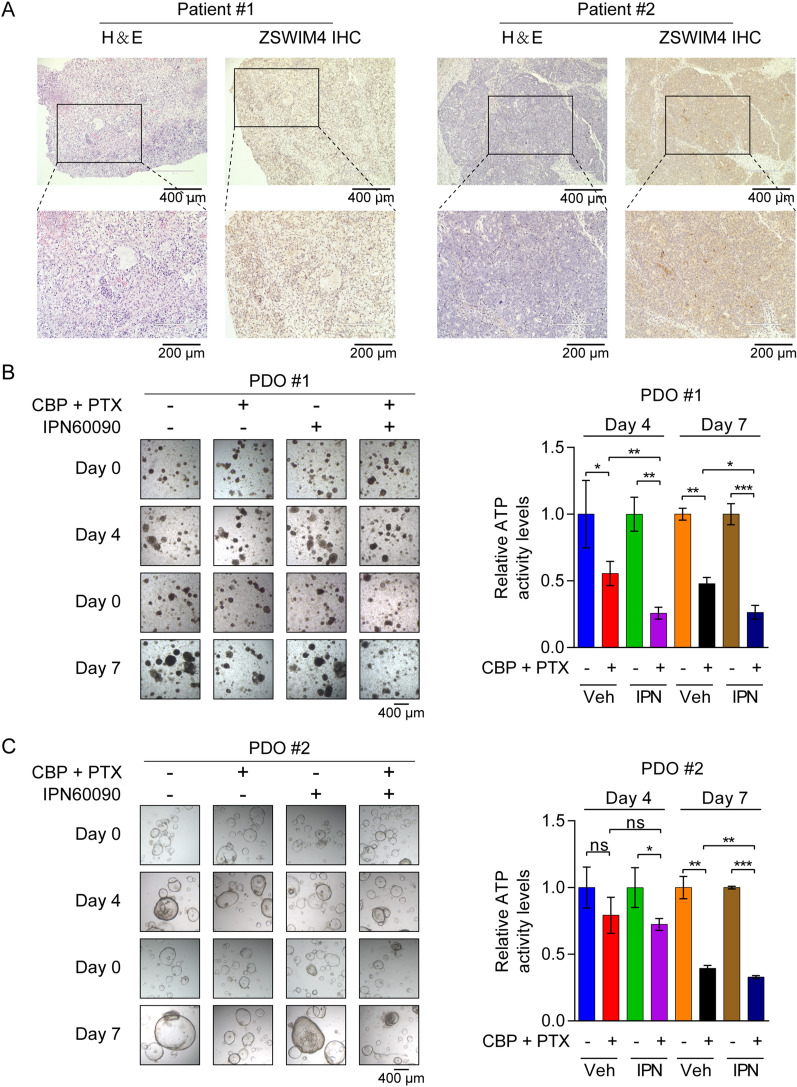
Patient-derived organoid (PDO) models with ZSWIM4 expression are sensitive to combined therapy. **A** Immunohistochemical analysis of ZSWIM4 protein expression in EOC tissues from two patients. **B**, **C** PDOs were treated with vehicle, CBP (100 μM) combined with PTX (10 nM), IPN60090 (50 μM), or CBP combined with PTX plus IPN60090, and the cell viability was measured on days four and seven using an ATP detection kit. PDO #1 **B**; PDO#2 **C**. Typical images (*left*) and statistical data (mean ± SD) (*right*). Veh, vehicle. IPN, IPN60090

CBP in combination with paclitaxel (PTX) is the first-line regimen for the clinical treatment of EOC [2]. We investigated whether the inhibition of ZSWIM4 could enhance the efficacy of this strategy. For this, we treated *ZSWIM4*-knockdown and control OVCAR8 cells with CBP (25 μM) alone or in combination with PTX (5 nM). Cell viability assays indicated that *ZSWIM4*-knockdown also sensitized EOC cells to this chemotherapy (Additional file 1: Fig. S8A). In both OVCAR8 and SKOV3 cells, the triple dosing regimen of CBP, PTX, and IPN60090 had an optimal inhibitory effect on cell viability (Additional file 1: Fig. S8B), and the use of ZSWIM4 inhibitors enhanced apoptosis induced by the combination of CBP and PTX (Additional file 1: Fig. S8C, D). PDO models are important preclinical models for evaluating drug responses and efficacy. To explore the potential clinical use of ZSWIM4-targeted inhibitors, tumor tissues exhibiting ZSWIM4 expression from two patients with EOC were obtained to construct PDO models (Fig. 8A). We then performed combination drug experiments using these two PDO models with the following treatments: vehicle, CBP (100 μM) combined with PTX (10 nM), IPN60090 (50 μM), and CBP combined with PTX plus IPN60090. Cell viability of the PDO models was measured on days four and seven using an adenosine triphosphate (ATP) detection kit. Notably, IPN60090 enhanced EOC cell sensitivity to chemotherapy in PDOs expressing ZSWIM4 (Fig. 8B, C). Collectively, these data suggest that therapeutic strategies targeting ZSWIM4 in patients with high expression of this marker may enhance the sensitivity of EOC cells to chemotherapy.

## Discussion

Chemotherapy resistance challenges the clinical treatment of EOC patients, and more than 60% of the patients are prone to recurrence and resistance to first-line chemotherapy drugs [37]. The development of chemoresistance in tumor cells is typically caused by abnormal transcriptional regulation patterns [38]. FOXK1 is an important transcription factor of aerobic glycolysis [39], which is closely associated with tumorigenesis, including triple negative breast cancer [40], gallbladder cancer [41], hepatocellular carcinoma [42], and ovarian malignancy [43]. A recent study reported that the Aurora-A/sex-determining region Y box-containing transcription factor 8 (SOX8)/FOXK1 axis enhances chemotherapy resistance in EOC cells and that inhibition of the transcription function of FOXK1 improves the therapeutic efficacy in platinum-resistant cells [29]. Hence, abnormal activation of FOXK1 facilitates the development or chemoresistance of EOC and could be regarded as a candidate therapeutic target against EOC. Indeed, clarifying the role of FOXK1 in EOC drug resistance will provide more favorable conditions for reducing chemotherapy resistance. Our study provided a novel mechanism of chemoresistance in which EOC cells survive in the presence of CBP by inducing ZSWIM4 expression via FOXK1-mediated transcription. However, the transcriptional activity of *ZSWIM4* was not fully lost after *FOXK1* knockdown, indicating that high expression of *ZSWIM4* in EOC was also regulated by other factors. Therefore, the molecular mechanisms underlying the upregulation of ZSWIM4 in EOC tumor tissues require further investigation.

The ZSWIM family proteins can interact with DNA or proteins and exert powerful regulatory effects on cells because of their unique SWIM domain [8]. Several studies have shown that ZSWIM5 is important in regulating neuronal development [44]. Recent studies have also reported that ZSWIM8 participates in protein ubiquitination as a ubiquitin ligase [10]. However, specific biological functions of ZSWIM4 have rarely been reported. In this study, through public database analysis and clinical sample validation, we observed a positive correlation between high ZSWIM4 expression and EOC recurrence and a restricted efficacy of platinum-based chemotherapy in patients with ZSWIM4 overexpression. In addition, ZSWIM4 inhibition exerts a significant chemo-sensitization effect in vivo and in vitro, as well as in PDO models, indicating that ZSWIM4 may serve as a therapeutic target and molecular marker for the prognosis of patients with EOC. By integrating bulk RNA-seq, signaling pathway analysis, and molecular and biochemical approaches, we found that *ZSWIM4* knockdown impacts glycine biosynthesis, increasing cellular ROS levels in EOC cells and sensitizing them to chemotherapeutic drugs. However, the mechanism through which ZSWIM4 regulates the expression of these key enzymes remains obscure. Notably, ZSWIM4 inhibition showed little impact on EOC cell proliferation, whereas the inhibition of glycine affected the growth of tumor cells, indicating that there is a compensatory mechanism in *ZSWIM4-*knockdown EOC cells to overcome this damage. Hence, a compensatory model associated with combined inhibition might have a killing effect on cells with high ZSWIM4 expression. Furthermore, we found that knockdown of *ZSWIM4* impacts the pentose phosphate pathway in EOC cells, further demonstrating the importance of ZSWIM4 in regulating EOC cell metabolism. Hence, further exploration of potential biological functions of ZSWIM4 in EOC will be carried out in the future.

Considering the lack of targeted inhibitors for ZSWIM4, we sought to identify small molecules from existing compound libraries that bind to, and potentially inhibit, ZSWIM4. The small-molecule compound IPN60090 is an orally active inhibitor of GLS-1, with no activity observed against GLS-2 [35]. GLS-1 inhibition alone or in combination with immune checkpoint blockade represents an effective therapeutic strategy for SWI/SNF-altered cancers such as *ARID1A*-mutated ovarian clear cell carcinoma [45, 46]. Here, we found that IPN60090 binds to ZSWIM4 and simulates the chemo-sensitization effect caused by ZSWIM4 knockdown. We will further elucidate how IPN60090 can be used to overcome chemotherapy resistance in EOC cells by altering intracellular metabolic processes in the following study. Subsequently, we also observed the activation of the FOXK1/ZSWIM4 axis and a significant synergistic effect between IPN60090 and CBP in SKOV3/CBP cells, suggesting that the resistance of SKOV3/CBP against CBP depends on the expression of ZSWIM4. Our study provides a compound framework for developing ZSWIM4 inhibitors. However, the ZSWIM4 inhibitor did not fully reverse the IC_50_ values of EOC cells to CBP, indicating the complexity of chemoresistance in EOC cells, which requires further investigation.

Due to the heterogeneity of EOC cells, the treatment responses and outcomes vary. Unfortunately, we are unable to fully evaluate the expression of ZSWIM4 in different pathological types of EOC. This implies that the TMA results can only suggest that ZSWIM4 expression may be higher in serous carcinoma than in clear cell carcinoma. Thus, to accurately predict the prognosis, we will expand the EOC sample set to evaluate ZSWIM4 protein expression in different EOC cell types. Overall, our study demonstrates the aberrant expression of ZSWIM4 in EOC and its involvement in chemosensitivity, suggesting that targeting ZSWIM4 could overcome EOC chemotherapy resistance.

## Conclusions

**Fig. 9:**
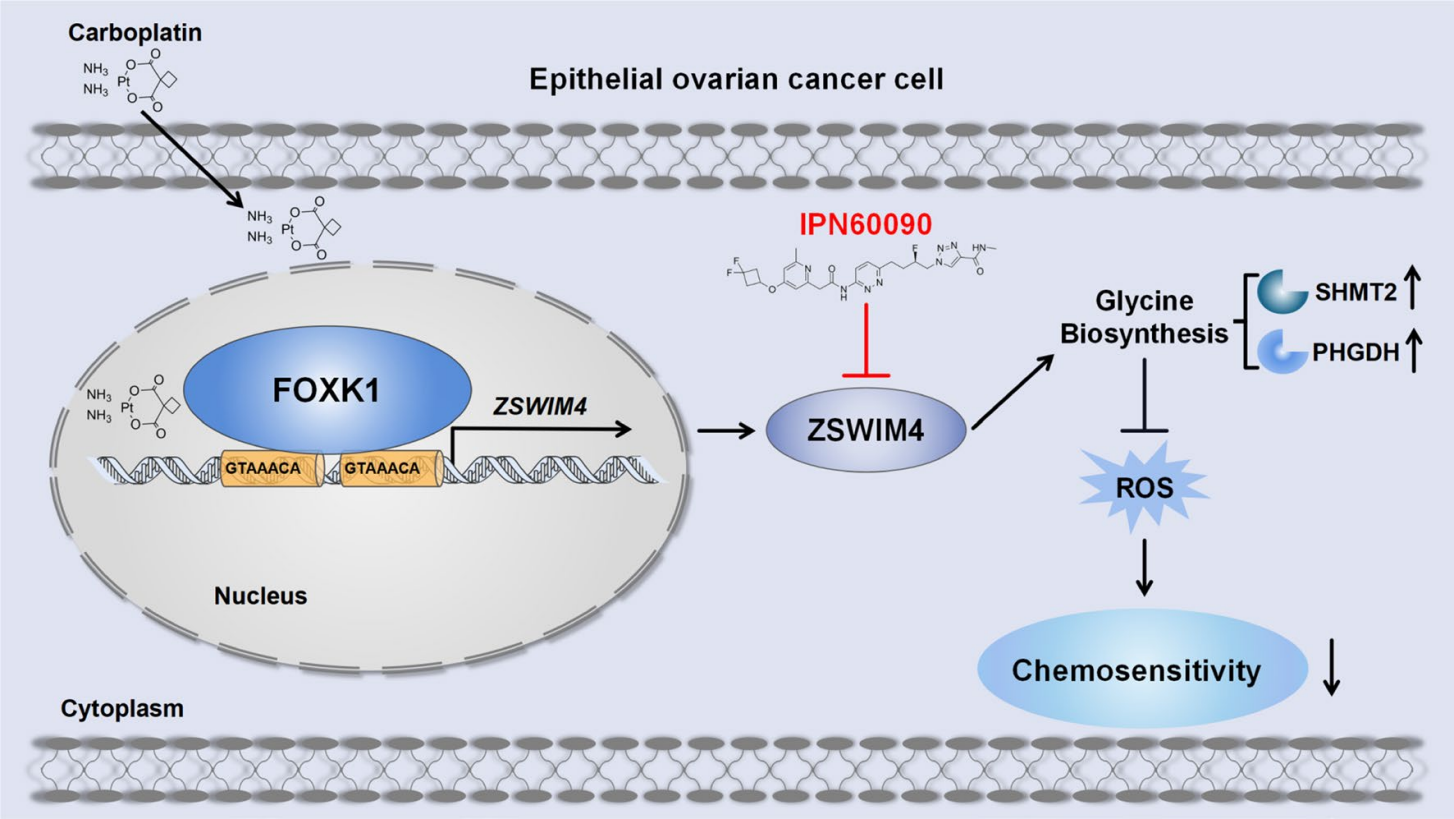
Graphic model of ZSWIM4 inhibition enhancing the chemosensitivity of EOC cells Epithelial ovarian cancer (EOC) cells survive in the presence of carboplatin by inducing ZSWIM4 expression via FOXK1-mediated transcription. Elevated ZSWIM4 levels upregulate the glycine biosynthesis-associated rate-limiting enzymes represented by SHMT2 and PHGDH, leading to increased intracellular glycine content and decreased levels of reactive oxygen species, further reducing the sensitivity of EOC cells to chemotherapy. IPN60090, a ZSWIM4-targeting inhibitor, enhances the chemosensitivity of EOC cells.

ZSWIM4 was overexpressed in EOC tumor tissues and was relevant to a poor prognosis. Furthermore, ZSWIM4 silencing promoted CBP-induced apoptosis and enhanced chemotherapy sensitivity in EOC cells. Moreover, ZSWIM4 was upregulated by FOXK1 following CBP treatment in EOC cells. Functionally, ZSWIM4 regulated the intracellular glycine metabolism to induce chemoresistance, and the ZSWIM4 inhibitor, IPN60090, sensitized EOC cells to chemotherapy by increasing cellular ROS levels induced by CBP (Fig. 9). These findings suggest that ZSWIM4 inhibition may be a promising treatment strategy for EOC.

### Supplementary Information

**Additional file 1. **Supplementary Figure 1-8 (Fig. S1-S8) and Supplementary Table 1-3 (Table S1-S3). **Figure S1**. ZSWIM4 is highly expressed in epithelial ovarian cancer (EOC) and indicates poor prognosis. **Figure S2**. Elevated ZSWIM4 resists chemotherapy in EOC cells. **Figure S3**. Silencing ZSWIM4 enhances the sensitivity of EOC cells to CBP. **Figure S4**. FOXK1-dependent ZSWIM4 transcription contributes to EOC cell chemotherapy resistance. **Figure S5**. Glycine metabolism reprogramming is triggered following ZSWIM4 knockdown in EOC cells. **Figure S6**. The ZSWIM4-targeting inhibitor, IPN60090 enhances chemotherapy sensitivity of EOC cells. **Figure S7**. The ZSWIM4 inhibitor enhances chemotherapy sensitivity in CBP-resistant SKOV3 cells. **Figure S8**. PDO models with ZSWIM4 expression are sensitive to the combined therapy. **Table S1**. Sequences used in this study. **Table S2**. Primers used in this study. **Table S3**. Gene sets enriched in OVCAR8-shCTR.

**Additional file 2. CUT&Tag sequencing results using the FOXK1 antibody in EOC cells.**

**Additional file 3. RNA sequencing results in OVCAR8-shCTR and OVCAR8-shZSWIM4 cells.**

**Additional file 4. Supplementary Table 4 (Table S4). The specific information of the patients involved in this study.**

## Data Availability

The datasets supporting the conclusions of this article are available in the GEPIA website, http://gepia.cancer-pku.cn/, the Kaplan–Meier plotter website, https://kmplot.com/analysis/, the UCSC Xena TCGA hub repository, https://xenabrowser.net/, and the GEO repository (code GSE45553). The CUT&Tag sequencing results using the FOXK1 antibody in EOC cells are presented in Additional file 2. The RNA sequencing results in OVCAR8-shCTR and OVCAR8-shZSWIM4 cells are presented in Additional file 3. The specific information of the patients involved in this study is presented in Additional file 4: Table S4. Further information and requests for reagents and resources should be directed to and will be made available by the corresponding authors upon reasonable request.

## Abbreviations

ANOVA: Analysis of variance
CBP: Carboplatin
EOC: Epithelial ovarian cancer
FBS: Fetal bovine serum
GEPIA: Gene expression profiling interactive analysis
GSEA: Gene set enrichment analysis
KEGG: Kyoto Encyclopedia of Genes and Genomes
OC: Ovarian cancer
PDO: Patient-derived organoid
PS: Penicillin–streptomycin
PTX: Paclitaxel
ROS: Reactive oxygen species
SD: Standard deviation
SPR: Surface plasmon resonance
TCGA: The cancer genome atlas
TNBC: Triple negative breast cancer
WT: Wild-type;
ZSWIM4: Zinc finger SWIM-type containing 4
PARP: Poly (ADP-ribose) polymerase
GLS-1: Glutaminase-1
