# Supplementary material for: ZSWIM4 inhibition improves chemosensitivity in epithelial ovarian cancer cells by suppressing intracellular glycine biosynthesis
Source: J Transl Med. 2024 Feb 21;22:192. doi: 10.1186/s12967-024-04980-8 (PMC10880229; doi:10.1186/s12967-024-04980-8)
Supplement: Supplementary file 1 — Additional file 1. Supplementary Figure 1-8 (Fig. S1-S8) and Supplementary Table 1-3 (Table S1-S3). Figure S1. ZSWIM4 is highly expressed in epithelial ovarian cancer (EOC) and indicates poor prognosis. Figure S2. Elevated ZSWIM4 resists chemotherapy in EOC cells. Figure S3. Silencing ZSWIM4 enhances the sensitivity of EOC cells to CBP. Figure S4. FOXK1-dependent ZSWIM4 transcription contributes to EOC cell chemotherapy resistance. Figure S5. Glycine metabolism reprogramming is triggered following ZSWIM4 knockdown in EOC cells. Figure S6. The ZSWIM4-targeting inhibitor, IPN60090 enhances chemotherapy sensitivity of EOC cells. Figure S7. The ZSWIM4 inhibitor enhances chemotherapy sensitivity in CBP-resistant SKOV3 cells. Figure S8. PDO models with ZSWIM4 expression are sensitive to the combined therapy. Table S1. Sequences used in this study. Table S2. Primers used in this study. Table S3. Gene sets enriched in OVCAR8-shCTR. [file 12967_2024_4980_MOESM1_ESM.docx]

Additional file information

ZSWIM4 inhibition improves chemosensitivity in epithelial ovarian cancer cells by suppressing intracellular glycine biosynthesis

Additional file Figures


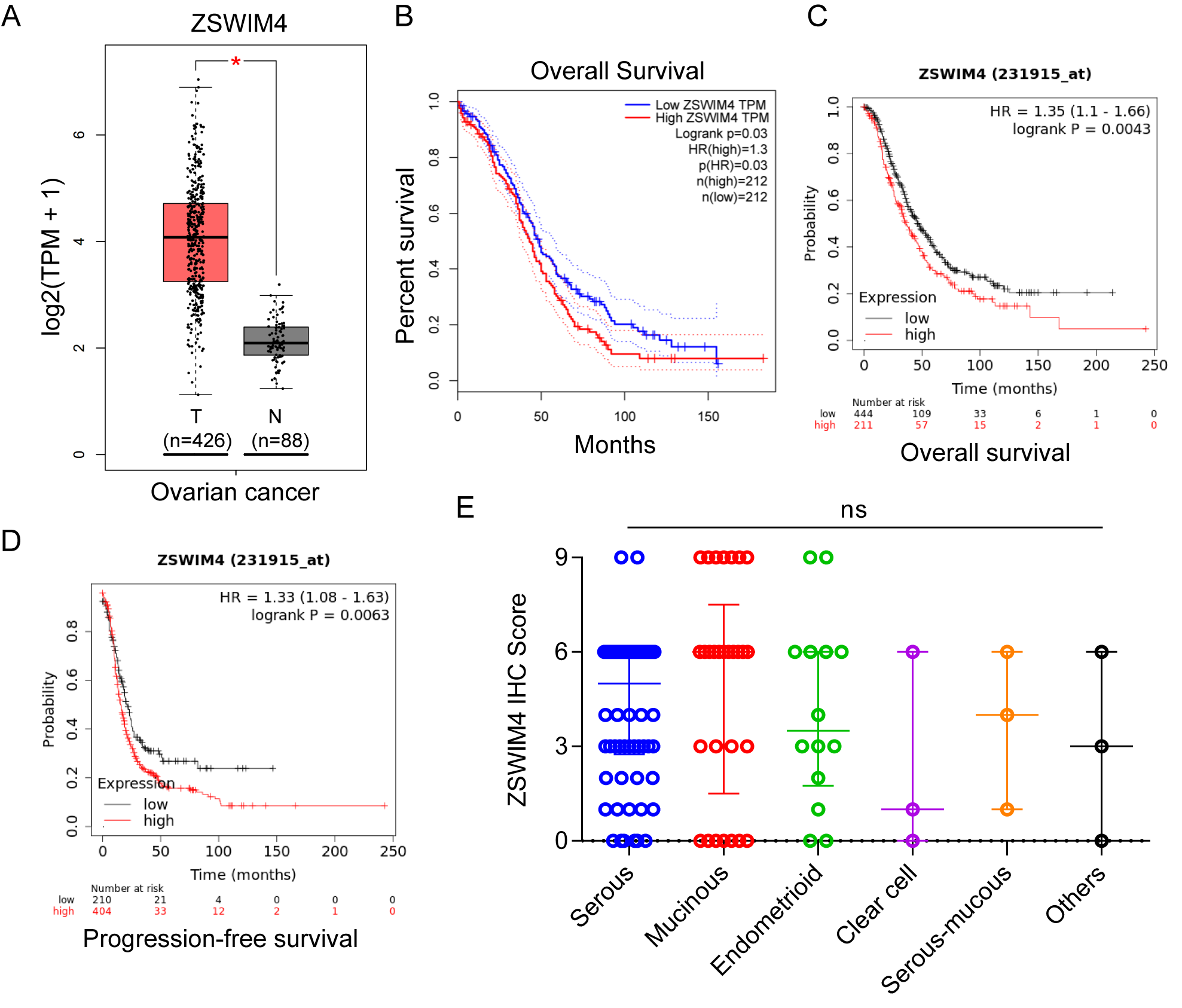


Figure S1. ZSWIM4 is highly expressed in epithelial ovarian cancer (EOC) and indicates poor prognosis. (A) *ZSWIM4* expression in ovarian tumors and normal tissues from the GEPIA database. (B) Survival analysis of ZSWIM4 in patients with ovarian cancer from the GEPIA database. (C, D) Survival analysis of *ZSWIM4* in patients with ovarian cancer from the Kaplan-Meier plotter website. Overall survival (C). Progression-free survival (D). (E) ZSWIM4 protein expression among different types of EOC based on the TMA.


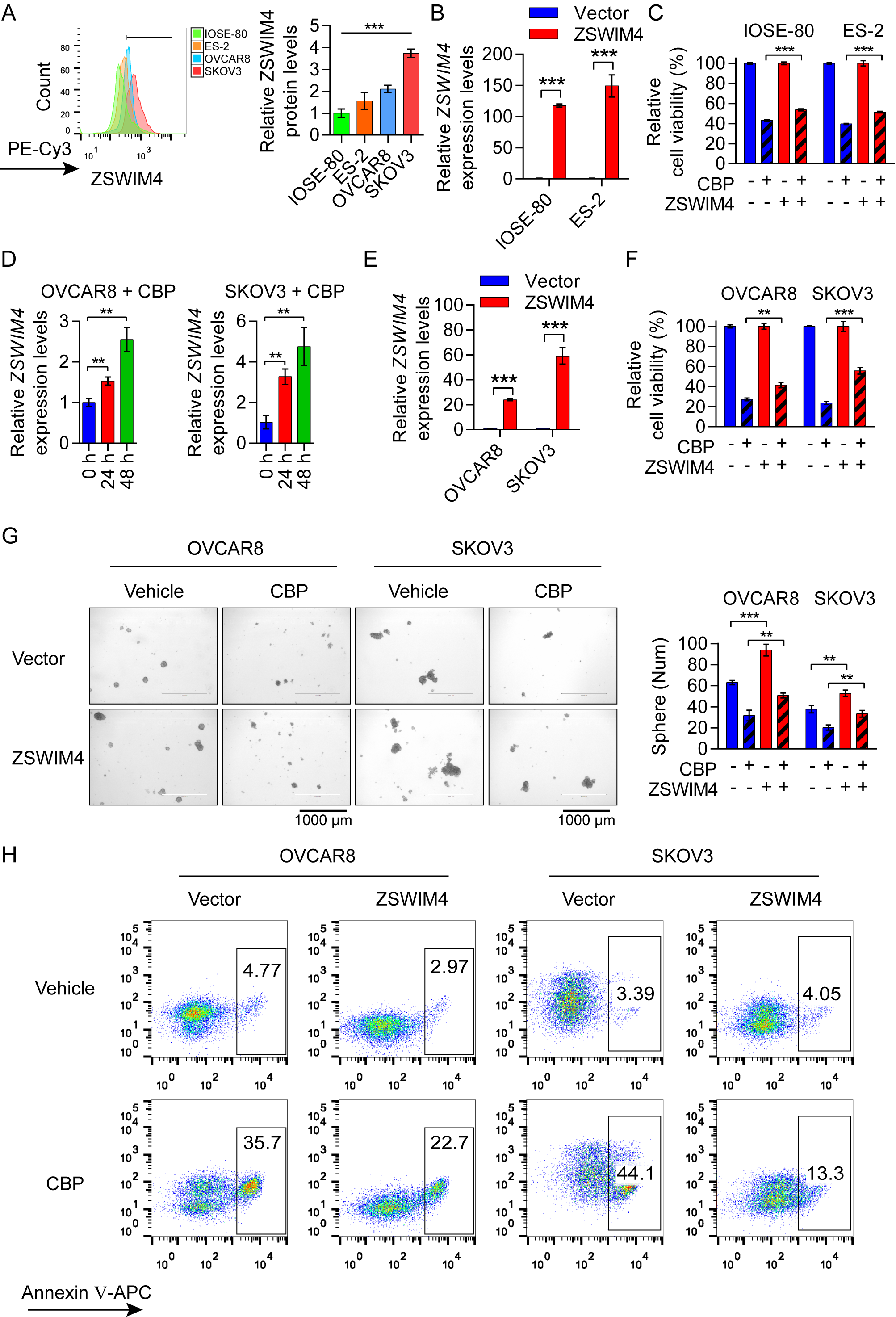


Figure S2. Elevated ZSWIM4 resists chemotherapy in EOC cells. (A) ZSWIM4 protein expression levels in IOSE-80, ES-2, SKOV3, and OVCAR8 cell lines. Representative flow cytometry histograms (*left*) and statistical data (mean ± SD) are shown (*right*). (B) *ZSWIM4* mRNA expression levels in the vector control and ZSWIM4-overexpressing IOSE-80 and ES-2 cells. (C) Vector control and ZSWIM4-overexpressing IOSE-80 and ES-2 cells incubated with vehicle control or CBP (50 μM) for 72 h. Cell viability was determined by Cell Counting Kit-8 (CCK-8) assay. (D) mRNA expression levels of *ZSWIM4* in OVCAR8 and SKOV3 cells treated with 75 μM CBP for 24 or 48 h. (E) *ZSWIM4* mRNA expression levels in the vector control and ZSWIM4-overexpressing OVCAR8 and SKOV3 cells. (F) Vector control and ZSWIM4-overexpressing OVCAR8 and SKOV3 cells incubated with vehicle control or CBP (100 μM) for 72 h. Cell viability was determined by CCK-8 assay. (G) Vector control and ZSWIM4-overexpressing OVCAR8 and SKOV3 cells were treated with vehicle control or CBP (50 μM) for 5 d. Representative tumorsphere images (*right*) and the statistical data (mean ± SD) (*left*) of tumorspheres are shown. (H) Representative flow cytometry results of ZSWIM4-overexpressing and vector control EOC cells with or without 50 μM CBP treatment for 72 h.


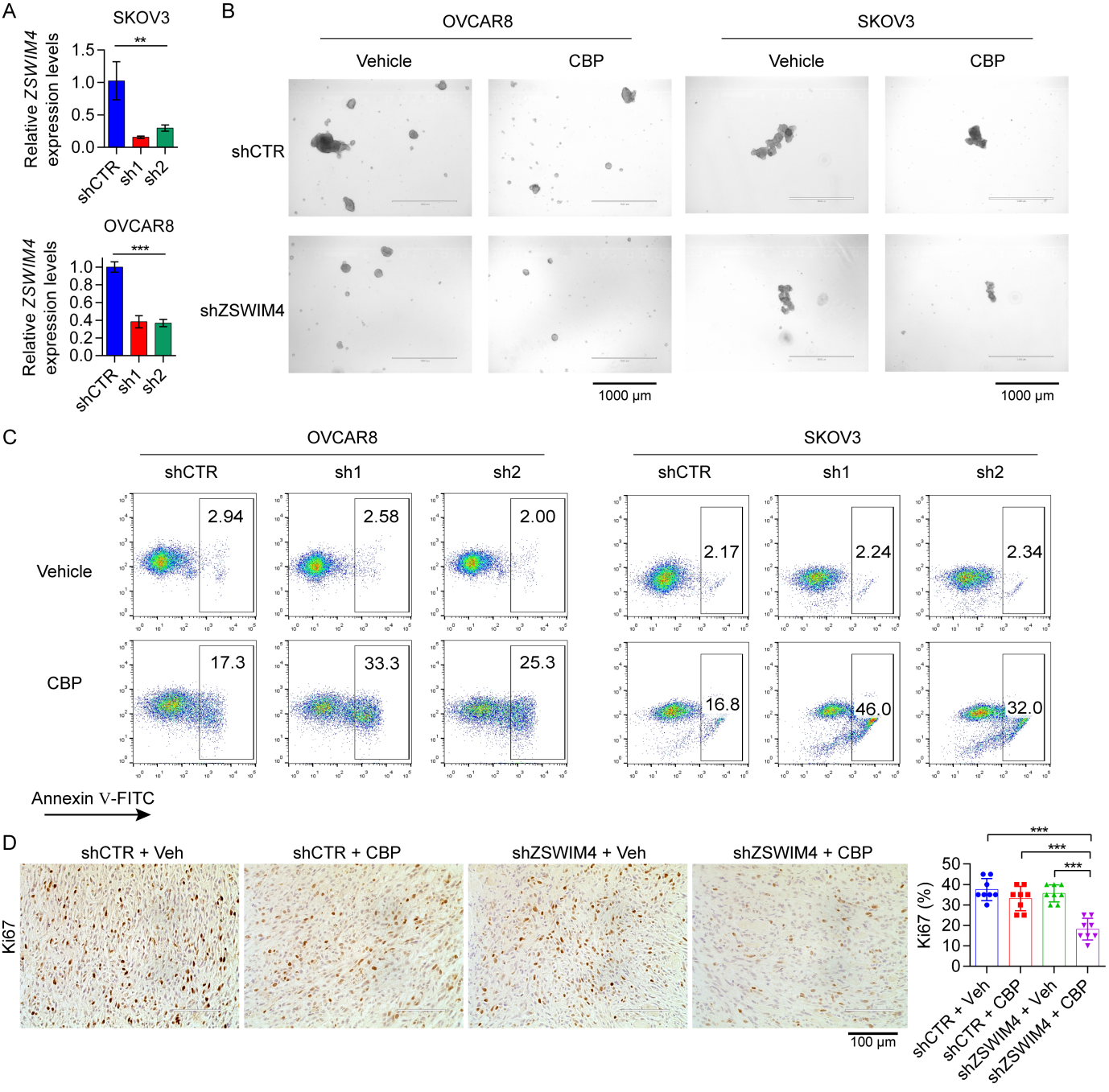


Figure S3. Silencing ZSWIM4 enhances the sensitivity of EOC cells to CBP. (A) *ZSWIM4* mRNA expression levels in vector control and *ZSWIM4*-knockdown EOC clones. (B) Representative images of tumorsphere formation in vector control and *ZSWIM4*-knockdown EOC cell clones with or without CBP (50 μM) treatment for five days. (C) Representative flow cytometry results of *ZSWIM4*-knockdown and their vector control cells treated with 50 μM CBP treatment for 72 h. (D) Representative graphs of Ki67 IHC staining of xenograft tumors (*left*) and statistical data (mean ± SD) (*right*) are presented.


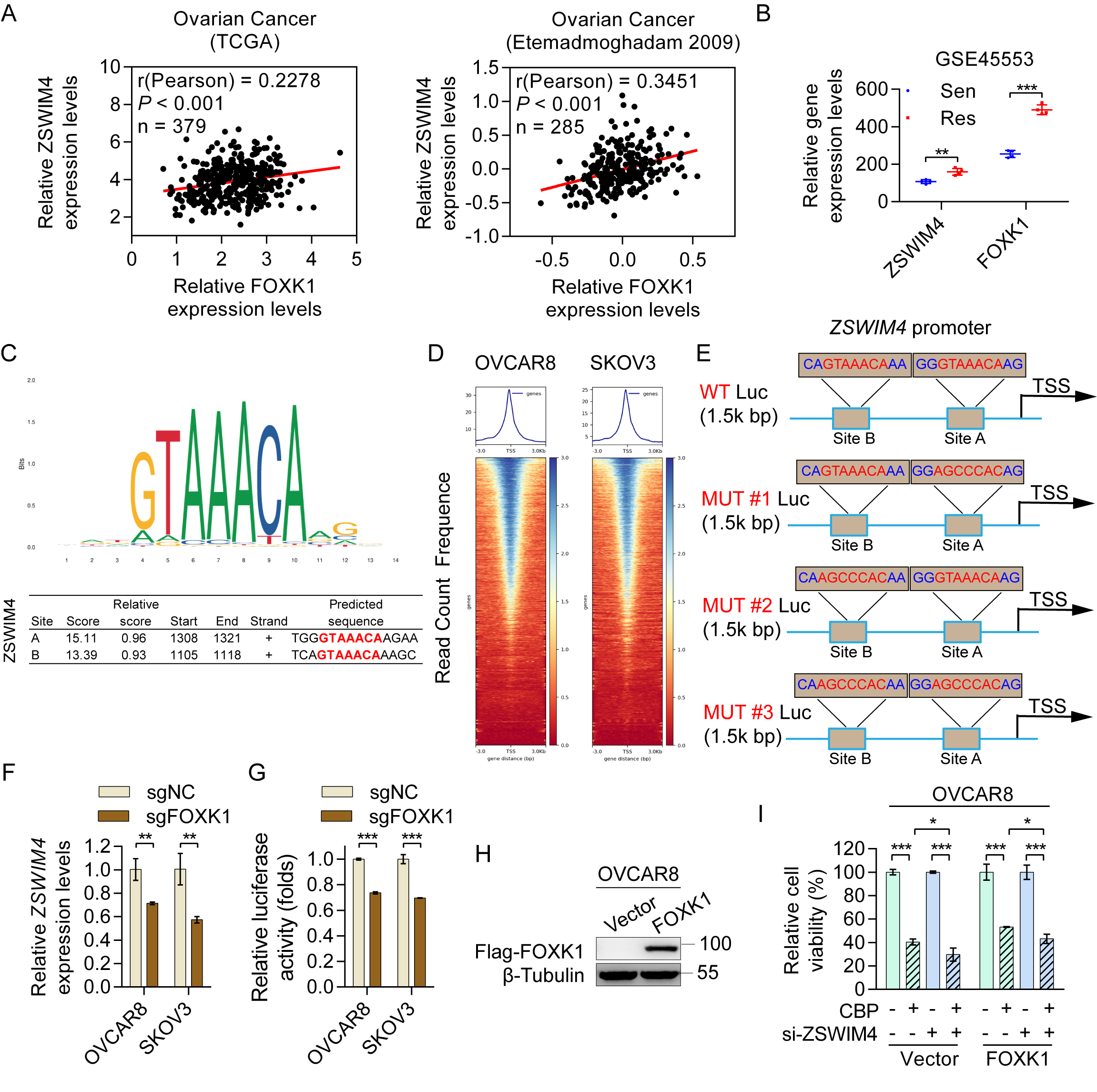


Figure S4. FOXK1-dependent *ZSWIM4* transcription contributes to EOC cell chemotherapy resistance. (A) Correlation between *ZSWIM4* and *FOXK1* analyzed in TCGA and Etemadmoghadam 2009 database. (B) *ZSWIM4* and *FOXK1* mRNA expression levels in GSE45553. (C) FOXK1 binding motif (*upper*) and predicted binding sites from the JASPAR website (*lower*). (D) Read count frequency of genomic regions in two EOC cell lines by CUT&Tag-seq. (E) The diagrammatic sketch of wild-type (WT) and potential FOXK1-binding sites mutant (MUT #1, MUT #2, and MUT #3) *ZSWIM4* promoter-luciferase plasmids. TSS, transcriptional start site. (F) *ZSWIM4* mRNA expression levels in *FOXK1*-knockdown EOC cells. (G) Transcriptional activity of *ZSWIM4* in FOXK1-knockdown EOC cells. (H) FOXK1 protein abundance in FOXK1-overexpressing OVCAR8 cells. (I) Silencing *ZSWIM4* in vector control and FOXK1-overexpressing OVCAR8 cells treated with vehicle or CBP (100 μM) for 72 h. Cell viability was assessed via the CCK-8 assay.


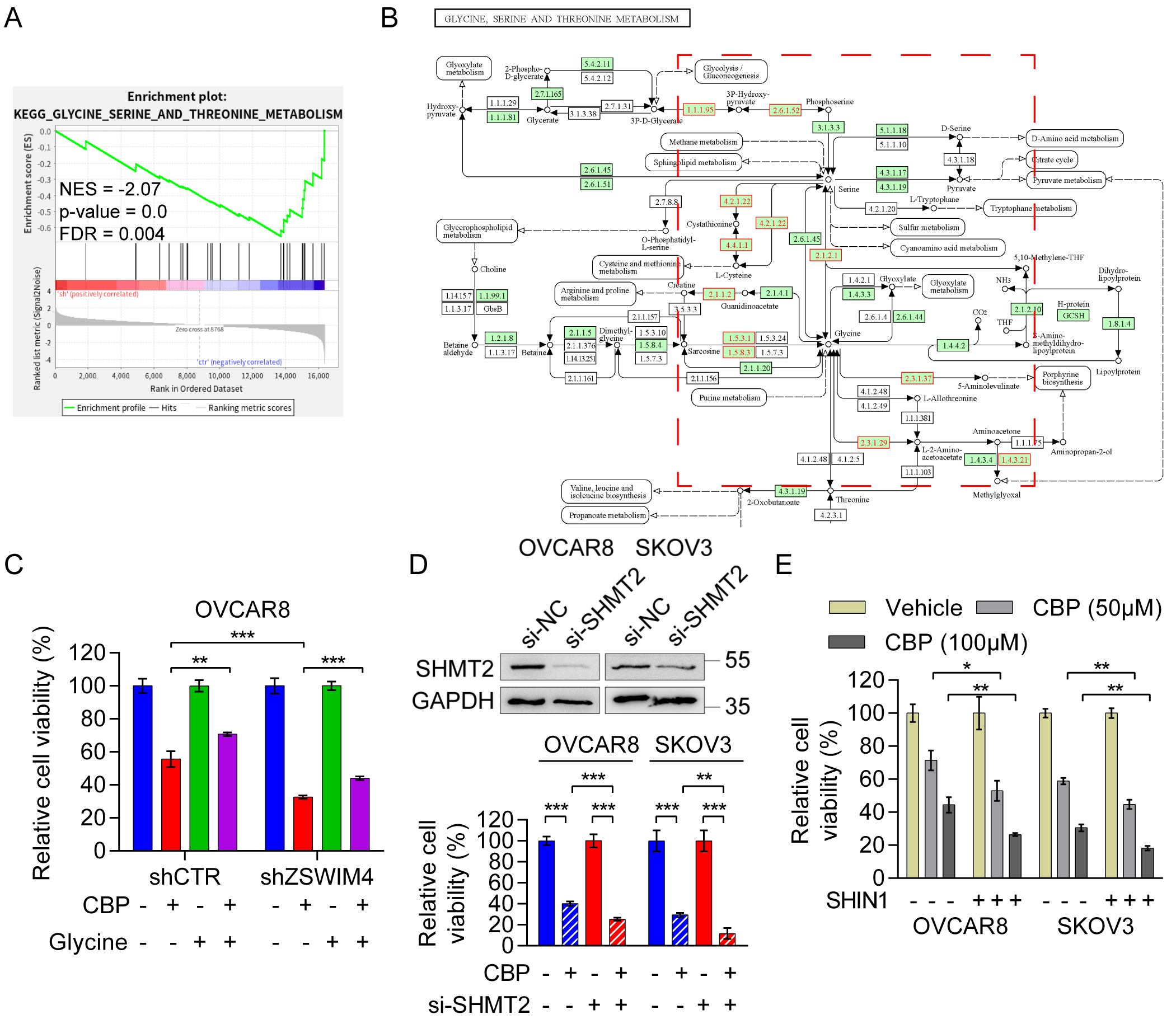


Figure S5. Glycine metabolism reprogramming is triggered following *ZSWIM4* knockdown in EOC cells. (A) GSEA analysis of correlations between *ZSWIM4* expression and the “GLYCINE_SERINE_AND_THREONINE_METABOLISM” pathway in OVCAR8 cells. (B) Downregulated genes involved in the biosynthesis of serine and glycine analyzed based on the Kyoto Encyclopedia of Genes and Genomes (KEGG) database. (C) Cell viability in vector control and *ZSWIM4*-knockdown OVCAR8 cells treated with 50 μM CBP in the presence or absence glycine (10 μM) for 72 h. (D) SHMT2 protein abundance in *SHMT2*-knockdown EOC cells (*upper*). Viability of *SHMT2*-knockdown EOC cells treated with 50 μM CBP for 72 h (*lower*). (E) Viability of OVCAR8 and SKOV3 cells treated with CBP (50 μM) or CBP (100 μM) in the presence or absence SHIN-1 (2.5 μM) for 72 h.


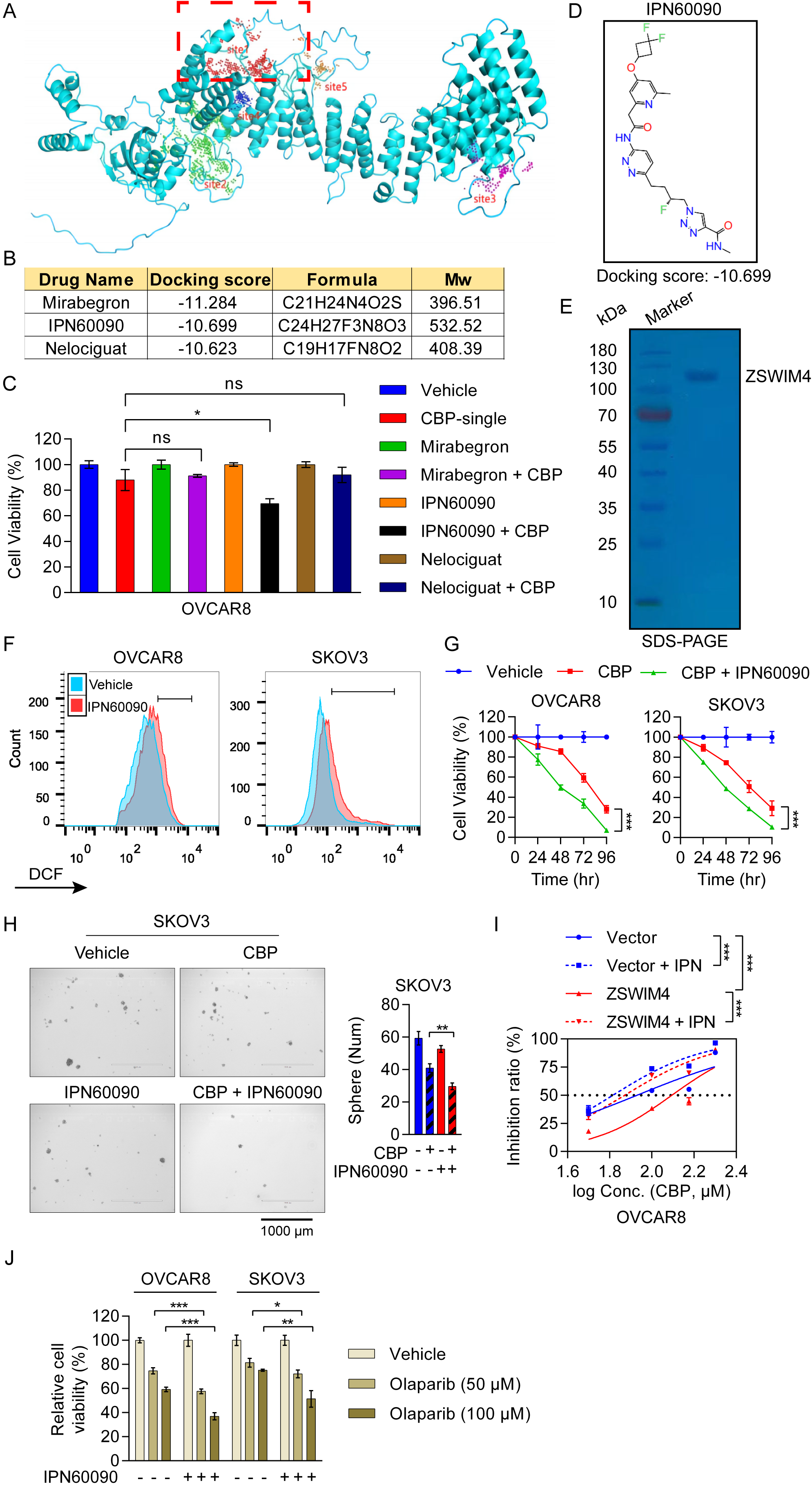


Figure S6. The ZSWIM4-targeting inhibitor, IPN60090 enhances chemotherapy sensitivity of EOC cells. (A) Site1 binding pocket in ZSWIM4 protein 3D structure. (B) Three small-molecule compounds with a docking score < ˗10.5. (C) CCK-8 assay analysis of chemosensitization effects of drugs. (D) Chemical structure of IPN60090. (E) The recombinant ZSWIM4 protein. (F) Representative flow cytometry histograms of two EOC cell lines treated with IPN60090 (10 μM) for 48 h. (G) Viability of EOC cells treated with vehicle, 50 μM CBP, or 50 μM CBP in combination with 10 μM IPN60090. (H) Tumorsphere formation in SKOV3 cells detected with CBP (50 μM) in the absence or presence of IPN60090 (10 μM). Representative images (*left*) and statistical data (mean ± SD) (*right*) are shown. (I) Drug sensitivity of CBP in ZSWIM4-overexpressing OVCAR8 cells with a gradient concentration of CBP in the presence or absence IPN60090 (10 μM) for 72 h. IPN, IPN60090. (J) Viability of OVCAR8 and SKOV3 cells treated with olaparib (50 μM) or olaparib (100 μM) in the presence or absence IPN60090 (50 μM) for 48 h.


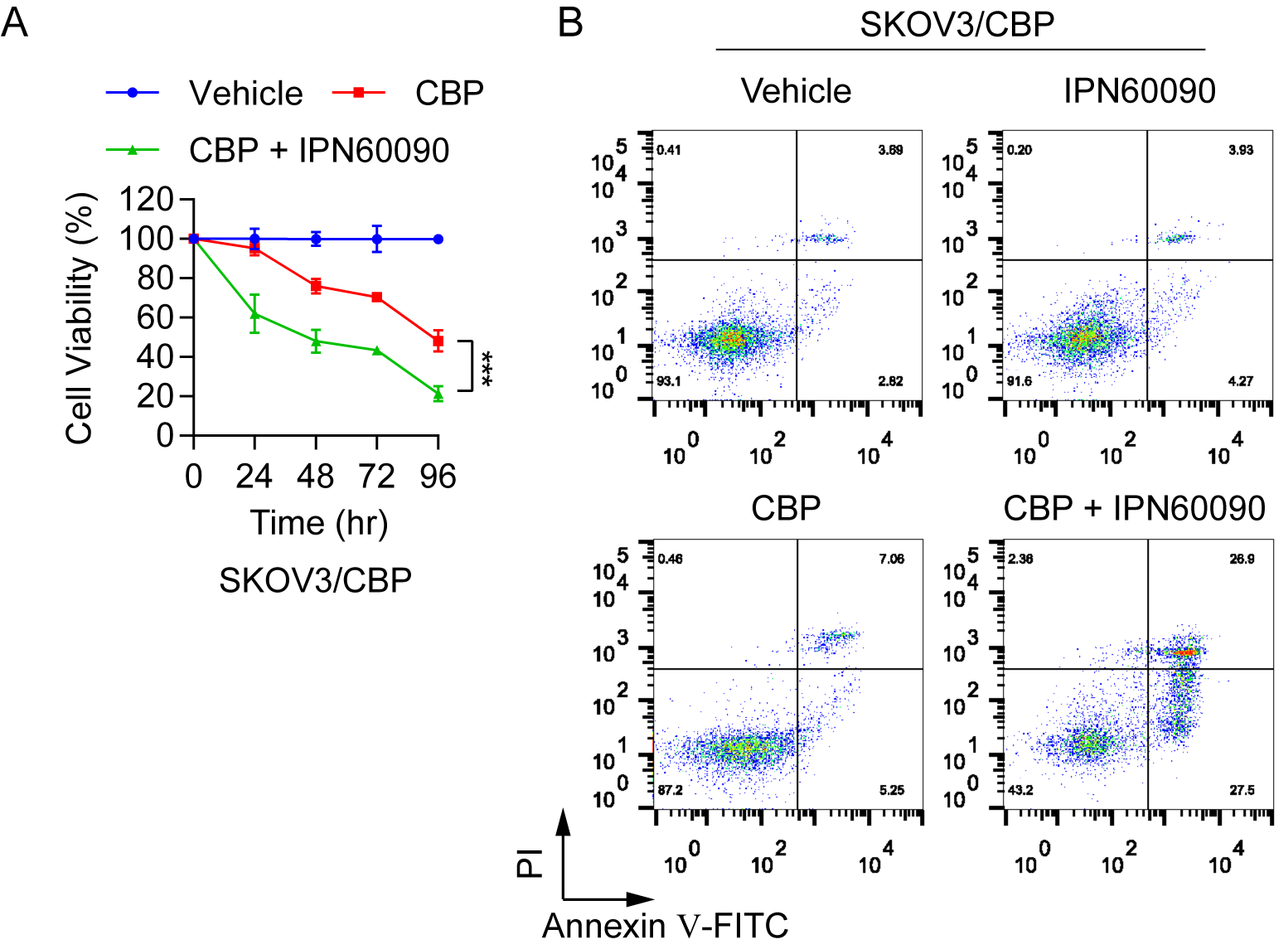


Figure S7. The ZSWIM4 inhibitor enhances chemotherapy sensitivity in CBP-resistant SKOV3 cells. (A) Cell viability in SKOV3/CBP cells treated with vehicle, 200 μM CBP, or 200 μM CBP in combination with 50 μM IPN60090. (B) Representative flow cytometry results of SKOV3/CBP treated with vehicle, 200 μM CBP, and/or 50 μM IPN60090 for 72 h.


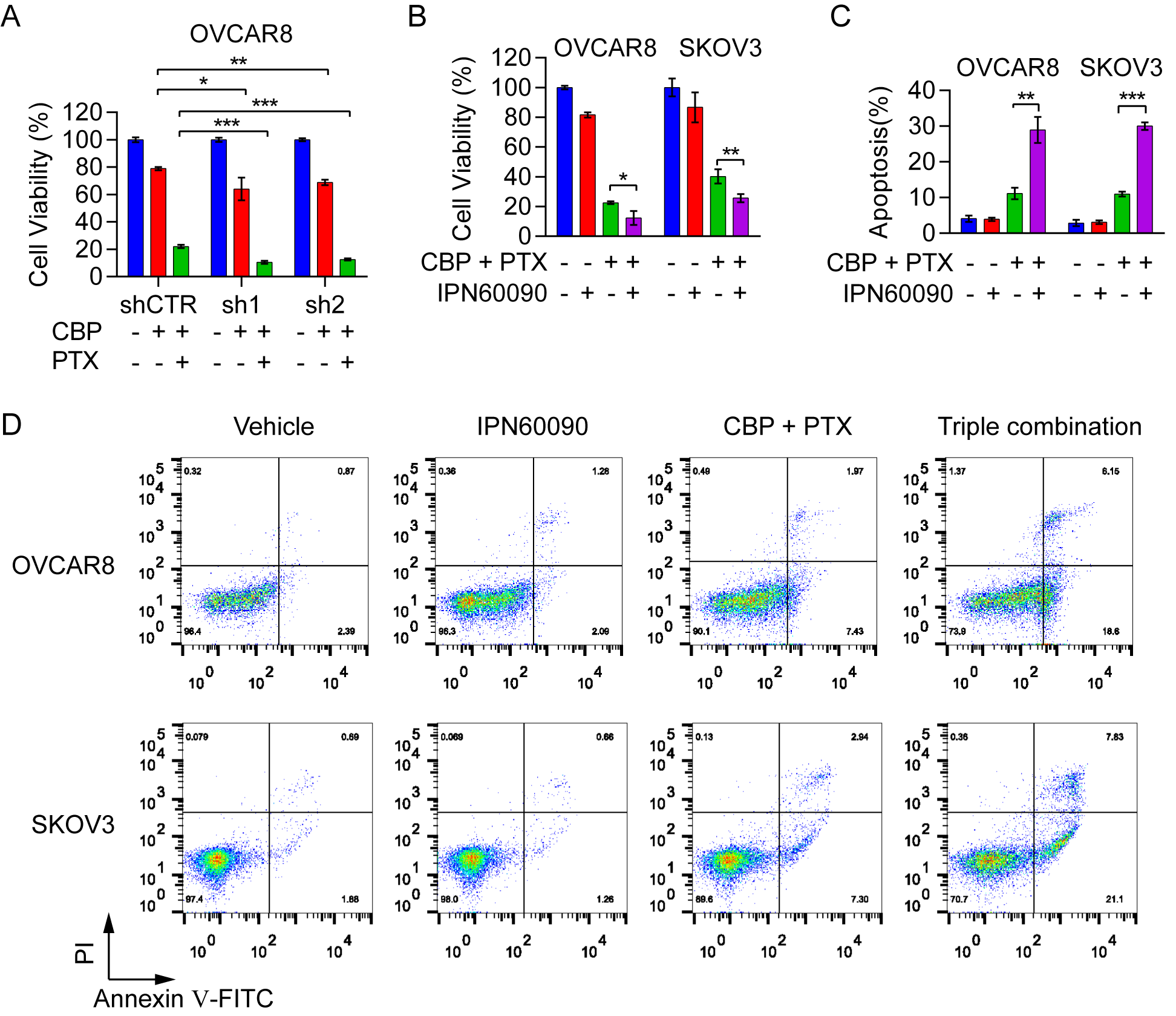


Figure S8. PDO models with ZSWIM4 expression are sensitive to the combined therapy. (A) Cell viability in *ZSWIM4*-knockdown and control OVCAR8 cells with CBP (25 μM) alone or in combination with PTX (5 nM) for 72 h. (B–D) OVCAR8 and SKOV3 cells treated with CBP (25 μM) in combination with PTX (5 nM) in the presence or absence IPN60090 (10 μM) for 72 h. Cell viability (B) and cell apoptosis rate (C) are presented as mean ± SD. Representative flow cytometry results are shown (D).

Supplementary Tables

Table S1. Sequences used in this study.

| Gene | Sequence (5′-3′) |
| --- | --- |
| shCTR | gatccgGCTTCGCGCCGTAGTCTTATCAAGAGTAAGACTACGGCGCGAAGCttttttg |
| sh-ZSWIM4-1 | gatccgCCCTTCTTGTATATTAAGGACTCAAGAGGTCCTTAATATACAAGAAGGGttttttg |
| sh-ZSWIM4-2 | gatccgGGATGACTCTGAACGTAATGATCAAGAGTCATTACGTTCAGAGTCATCCttttttg |
| si-NC | UUUUCCGAACGUGUCAGGUTT |
| si-SHMT2 | CUGGCCUCAUUGACUACAATT |
| si-ZSWIM4 | GCGUGUUGCAAGUGGGAUUTT |
| sg-FOXK1 | caccgTTGCGGCCGATGGTGACGCT |

Table S2. Primers used in this study.

| Gene | Forward primer | Reverse primer |
| --- | --- | --- |
| ZSWIM4 | 5′-CTACCTGTTCACCGCACTG-3′ | 5′-ATGGGCAGCCTCATAGCTC-3′ |
| GAPDH | 5′-TCTGACTTCAACAGCGACAC-3′ | 5′-CGTTGTCATACCAGGAAATGAG-3′ |
| SARDH | 5′-TACTACGGGGCTTACGGGAG-3′ | 5′-AGGCACTCCTTCTTGATCGTG-3′ |
| PHGDH | 5′-ACGTGTTTACGGAAGAGCCG-3′ | 5′-CCCTTCACCATGTCCACGAA-3′ |
| PSAT1 | 5′-TGAAAGGGCATAGGTCTGTGG-3′ | 5′-CTGGTTAGGATGTGTTCATAGCTGA-3′ |
| SHMT2 | 5′-CCGCATGAGAGAGGTGTGT-3′ | 5′-GCTTGAAAGGCGAGGGAATC-3′ |
| PIPOX | 5′-CACGCACAGACATCGGAGA-3′ | 5′-TGCTCATCAGGGGTATTCGTG-3′ |
| GCAT | 5′-AACTTCTGTGCCAACAACTACC-3′ | 5′-TTCTTGTGGATGCTCTGGGTTC-3′ |
| ZSWIM4  (ChIP-qPCR) | 5′-CCCCGAAACTCCGCATAAGG-3′ | 5′-GTTTACCCAACCAGAGCGCA-3′ |

Table S3. Gene sets enriched in OVCAR8-shCTR

| Rank | GS follow link to MSigDB | NES | NOM p-val | FDR q-val |
| --- | --- | --- | --- | --- |
| 1 | KEGG_GLYCINE_SERINE_AND_THREONINE_METABOLISM | -2.07 | 0 | 0.004 |
| 2 | KEGG_CARDIAC_MUSCLE_CONTRACTION | -1.79 | 0 | 0.092 |
| 3 | KEGG_PENTOSE_PHOSPHATE_PATHWAY | -1.7 | 0.011 | 0.154 |
| 4 | KEGG_NEUROACTIVE_LIGAND_RECEPTOR_INTERACTION | -1.68 | 0.002 | 0.133 |
| 5 | KEGG_HYPERTROPHIC_CARDIOMYOPATHY_HCM | -1.67 | 0.004 | 0.126 |
| 6 | KEGG_CALCIUM_SIGNALING_PATHWAY | -1.65 | 0 | 0.127 |
| 7 | KEGG_ECM_RECEPTOR_INTERACTION | -1.64 | 0.004 | 0.116 |
| 8 | KEGG_DILATED_CARDIOMYOPATHY | -1.63 | 0 | 0.115 |
| 9 | KEGG_TYPE_II_DIABETES_MELLITUS | -1.62 | 0.012 | 0.108 |
| 10 | KEGG_NOTCH_SIGNALING_PATHWAY | -1.61 | 0.008 | 0.104 |
| 11 | KEGG_STEROID_BIOSYNTHESIS | -1.61 | 0.02 | 0.095 |
| 12 | KEGG_CELL_ADHESION_MOLECULES_CAMS | -1.6 | 0 | 0.096 |
| 13 | KEGG_AXON_GUIDANCE | -1.58 | 0.002 | 0.112 |
| 14 | KEGG_ENDOCYTOSIS | -1.53 | 0.003 | 0.157 |
| 15 | KEGG_MAPK_SIGNALING_PATHWAY | -1.49 | 0.002 | 0.199 |
| 16 | KEGG_GLYCOSAMINOGLYCAN_BIOSYNTHESIS_CHONDROITIN_SULFATE | -1.49 | 0.051 | 0.193 |
| 17 | KEGG_FRUCTOSE_AND_MANNOSE_METABOLISM | -1.48 | 0.034 | 0.19 |
| 18 | KEGG_BLADDER_CANCER | -1.48 | 0.03 | 0.181 |
| 19 | KEGG_ADIPOCYTOKINE_SIGNALING_PATHWAY | -1.47 | 0.021 | 0.192 |
| 20 | KEGG_LEUKOCYTE_TRANSENDOTHELIAL_MIGRATION | -1.46 | 0.012 | 0.195 |
| 21 | KEGG_GALACTOSE_METABOLISM | -1.44 | 0.053 | 0.21 |
| 22 | KEGG_PHENYLALANINE_METABOLISM | -1.44 | 0.074 | 0.201 |
| 23 | KEGG_ALDOSTERONE_REGULATED_SODIUM_REABSORPTION | -1.44 | 0.044 | 0.199 |
| 24 | KEGG_FOCAL_ADHESION | -1.41 | 0.012 | 0.24 |
| 25 | KEGG_MATURITY_ONSET_DIABETES_OF_THE_YOUNG | -1.4 | 0.102 | 0.24 |
| 26 | KEGG_TASTE_TRANSDUCTION | -1.4 | 0.054 | 0.235 |
| 27 | KEGG_AMYOTROPHIC_LATERAL_SCLEROSIS_ALS | -1.39 | 0.065 | 0.235 |
| 28 | KEGG_MELANOGENESIS | -1.38 | 0.036 | 0.239 |
| 29 | KEGG_BASAL_CELL_CARCINOMA | -1.38 | 0.053 | 0.232 |
| 30 | KEGG_PRION_DISEASES | -1.37 | 0.086 | 0.243 |
| 31 | KEGG_BETA_ALANINE_METABOLISM | -1.34 | 0.119 | 0.295 |
| 32 | KEGG_ARGININE_AND_PROLINE_METABOLISM | -1.31 | 0.112 | 0.341 |
| 33 | KEGG_REGULATION_OF_ACTIN_CYTOSKELETON | -1.31 | 0.03 | 0.332 |
| 34 | KEGG_VEGF_SIGNALING_PATHWAY | -1.31 | 0.081 | 0.332 |
| 35 | KEGG_PPAR_SIGNALING_PATHWAY | -1.29 | 0.092 | 0.362 |
| 36 | KEGG_GLYCEROLIPID_METABOLISM | -1.28 | 0.119 | 0.37 |
| 37 | KEGG_FC_GAMMA_R_MEDIATED_PHAGOCYTOSIS | -1.26 | 0.082 | 0.408 |
| 38 | KEGG_GLYCOSPHINGOLIPID_BIOSYNTHESIS_LACTO_AND_NEOLACTO_SERIES | -1.25 | 0.166 | 0.418 |
| 39 | KEGG_HEDGEHOG_SIGNALING_PATHWAY | -1.25 | 0.122 | 0.412 |
| 40 | KEGG_TIGHT_JUNCTION | -1.24 | 0.1 | 0.428 |
| 41 | KEGG_COMPLEMENT_AND_COAGULATION_CASCADES | -1.23 | 0.142 | 0.429 |
| 42 | KEGG_VASCULAR_SMOOTH_MUSCLE_CONTRACTION | -1.22 | 0.14 | 0.446 |
| 43 | KEGG_TYROSINE_METABOLISM | -1.2 | 0.212 | 0.485 |
| 44 | KEGG_ALANINE_ASPARTATE_AND_GLUTAMATE_METABOLISM | -1.2 | 0.208 | 0.486 |
| 45 | KEGG_HUNTINGTONS_DISEASE | -1.19 | 0.098 | 0.481 |
| 46 | KEGG_SYSTEMIC_LUPUS_ERYTHEMATOSUS | -1.19 | 0.144 | 0.473 |
| 47 | KEGG_VASOPRESSIN_REGULATED_WATER_REABSORPTION | -1.19 | 0.192 | 0.465 |
| 48 | KEGG_FC_EPSILON_RI_SIGNALING_PATHWAY | -1.18 | 0.204 | 0.499 |
| 49 | KEGG_AUTOIMMUNE_THYROID_DISEASE | -1.17 | 0.237 | 0.493 |
| 50 | KEGG_LYSOSOME | -1.16 | 0.156 | 0.508 |
| 51 | KEGG_INSULIN_SIGNALING_PATHWAY | -1.16 | 0.178 | 0.507 |
| 52 | KEGG_PATHOGENIC_ESCHERICHIA_COLI_INFECTION | -1.15 | 0.236 | 0.517 |
| 53 | KEGG_GLYCOSAMINOGLYCAN_DEGRADATION | -1.13 | 0.288 | 0.563 |
| 54 | KEGG_RNA_POLYMERASE | -1.12 | 0.317 | 0.596 |
| 55 | KEGG_ALLOGRAFT_REJECTION | -1.11 | 0.294 | 0.609 |
| 56 | KEGG_GNRH_SIGNALING_PATHWAY | -1.1 | 0.269 | 0.619 |
| 57 | KEGG_WNT_SIGNALING_PATHWAY | -1.09 | 0.26 | 0.633 |
| 58 | KEGG_GLYCOLYSIS_GLUCONEOGENESIS | -1.08 | 0.31 | 0.657 |
| 59 | KEGG_ONE_CARBON_POOL_BY_FOLATE | -1.07 | 0.359 | 0.687 |
| 60 | KEGG_GRAFT_VERSUS_HOST_DISEASE | -1.06 | 0.372 | 0.686 |
| 61 | KEGG_NEUROTROPHIN_SIGNALING_PATHWAY | -1.06 | 0.305 | 0.683 |
| 62 | KEGG_ADHERENS_JUNCTION | -1.06 | 0.354 | 0.674 |
| 63 | KEGG_LEISHMANIA_INFECTION | -1.05 | 0.368 | 0.7 |
| 64 | KEGG_THYROID_CANCER | -1.04 | 0.374 | 0.704 |
| 65 | KEGG_PROXIMAL_TUBULE_BICARBONATE_RECLAMATION | -1.04 | 0.415 | 0.717 |
| 66 | KEGG_STEROID_HORMONE_BIOSYNTHESIS | -1.04 | 0.375 | 0.707 |
| 67 | KEGG_LONG_TERM_POTENTIATION | -1.03 | 0.41 | 0.726 |
| 68 | KEGG_ALZHEIMERS_DISEASE | -1.01 | 0.433 | 0.769 |
| 69 | KEGG_DRUG_METABOLISM_OTHER_ENZYMES | -1.01 | 0.46 | 0.759 |
| 70 | KEGG_CHEMOKINE_SIGNALING_PATHWAY | -1.01 | 0.44 | 0.749 |
| 71 | KEGG_CYTOKINE_CYTOKINE_RECEPTOR_INTERACTION | -1.01 | 0.432 | 0.739 |
| 72 | KEGG_GLYCEROPHOSPHOLIPID_METABOLISM | -1.01 | 0.439 | 0.735 |
| 73 | KEGG_HEMATOPOIETIC_CELL_LINEAGE | -1 | 0.419 | 0.729 |
| 74 | KEGG_AMINOACYL_TRNA_BIOSYNTHESIS | -1 | 0.433 | 0.72 |
| 75 | KEGG_VIRAL_MYOCARDITIS | -1 | 0.451 | 0.716 |
| 76 | KEGG_INOSITOL_PHOSPHATE_METABOLISM | -0.99 | 0.448 | 0.734 |
| 77 | KEGG_GLYCOSAMINOGLYCAN_BIOSYNTHESIS_HEPARAN_SULFATE | -0.98 | 0.488 | 0.757 |
| 78 | KEGG_TRYPTOPHAN_METABOLISM | -0.98 | 0.501 | 0.762 |
| 79 | KEGG_B_CELL_RECEPTOR_SIGNALING_PATHWAY | -0.97 | 0.536 | 0.783 |
| 80 | KEGG_PYRIMIDINE_METABOLISM | -0.96 | 0.541 | 0.784 |
| 81 | KEGG_NATURAL_KILLER_CELL_MEDIATED_CYTOTOXICITY | -0.96 | 0.499 | 0.782 |
| 82 | KEGG_NICOTINATE_AND_NICOTINAMIDE_METABOLISM | -0.96 | 0.524 | 0.776 |
| 83 | KEGG_PATHWAYS_IN_CANCER | -0.96 | 0.603 | 0.771 |
| 84 | KEGG_NITROGEN_METABOLISM | -0.93 | 0.541 | 0.833 |
| 85 | KEGG_GLYCOSPHINGOLIPID_BIOSYNTHESIS_GANGLIO_SERIES | -0.93 | 0.54 | 0.83 |
| 86 | KEGG_DORSO_VENTRAL_AXIS_FORMATION | -0.93 | 0.556 | 0.821 |
| 87 | KEGG_LINOLEIC_ACID_METABOLISM | -0.93 | 0.575 | 0.818 |
| 88 | KEGG_GLYOXYLATE_AND_DICARBOXYLATE_METABOLISM | -0.91 | 0.578 | 0.838 |
| 89 | KEGG_PARKINSONS_DISEASE | -0.91 | 0.643 | 0.832 |
| 90 | KEGG_ERBB_SIGNALING_PATHWAY | -0.91 | 0.638 | 0.834 |
| 91 | KEGG_TOLL_LIKE_RECEPTOR_SIGNALING_PATHWAY | -0.89 | 0.687 | 0.872 |
| 92 | KEGG_SELENOAMINO_ACID_METABOLISM | -0.88 | 0.636 | 0.881 |
| 93 | KEGG_CITRATE_CYCLE_TCA_CYCLE | -0.87 | 0.652 | 0.903 |
| 94 | KEGG_LONG_TERM_DEPRESSION | -0.87 | 0.702 | 0.905 |
| 95 | KEGG_PORPHYRIN_AND_CHLOROPHYLL_METABOLISM | -0.86 | 0.674 | 0.91 |
| 96 | KEGG_ANTIGEN_PROCESSING_AND_PRESENTATION | -0.86 | 0.719 | 0.902 |
| 97 | KEGG_ARACHIDONIC_ACID_METABOLISM | -0.86 | 0.716 | 0.896 |
| 98 | KEGG_ABC_TRANSPORTERS | -0.86 | 0.709 | 0.89 |
| 99 | KEGG_BIOSYNTHESIS_OF_UNSATURATED_FATTY_ACIDS | -0.84 | 0.694 | 0.928 |
| 100 | KEGG_MTOR_SIGNALING_PATHWAY | -0.82 | 0.765 | 0.959 |
| 101 | KEGG_HISTIDINE_METABOLISM | -0.8 | 0.775 | 0.973 |
| 102 | KEGG_JAK_STAT_SIGNALING_PATHWAY | -0.8 | 0.889 | 0.972 |
| 103 | KEGG_PHOSPHATIDYLINOSITOL_SIGNALING_SYSTEM | -0.8 | 0.859 | 0.965 |
| 104 | KEGG_BUTANOATE_METABOLISM | -0.8 | 0.78 | 0.957 |
| 105 | KEGG_TYPE_I_DIABETES_MELLITUS | -0.79 | 0.778 | 0.955 |
| 106 | KEGG_ACUTE_MYELOID_LEUKEMIA | -0.78 | 0.874 | 0.966 |
| 107 | KEGG_OXIDATIVE_PHOSPHORYLATION | -0.78 | 0.918 | 0.957 |
| 108 | KEGG_O_GLYCAN_BIOSYNTHESIS | -0.77 | 0.82 | 0.962 |
| 109 | KEGG_AMINO_SUGAR_AND_NUCLEOTIDE_SUGAR_METABOLISM | -0.77 | 0.859 | 0.961 |
| 110 | KEGG_ENDOMETRIAL_CANCER | -0.77 | 0.884 | 0.953 |
| 111 | KEGG_LYSINE_DEGRADATION | -0.75 | 0.888 | 0.973 |
| 112 | KEGG_CYTOSOLIC_DNA_SENSING_PATHWAY | -0.74 | 0.885 | 0.966 |
| 113 | KEGG_ARRHYTHMOGENIC_RIGHT_VENTRICULAR_CARDIOMYOPATHY_ARVC | -0.73 | 0.921 | 0.974 |
| 114 | KEGG_T_CELL_RECEPTOR_SIGNALING_PATHWAY | -0.71 | 0.983 | 0.982 |
| 115 | KEGG_RETINOL_METABOLISM | -0.7 | 0.927 | 0.982 |
| 116 | KEGG_CYSTEINE_AND_METHIONINE_METABOLISM | -0.68 | 0.924 | 0.985 |
| 117 | KEGG_OLFACTORY_TRANSDUCTION | -0.64 | 0.991 | 1 |
| 118 | KEGG_FATTY_ACID_METABOLISM | -0.63 | 0.966 | 0.995 |
| 119 | KEGG_ASTHMA | -0.62 | 0.929 | 0.99 |
| 120 | KEGG_PRIMARY_IMMUNODEFICIENCY | -0.56 | 0.981 | 0.994 |
